# Melan-Dx: a knowledge-enhanced vision-language framework improves differential diagnosis of melanocytic neoplasm pathology

**DOI:** 10.1038/s41746-026-02357-3

**Published:** 2026-01-20

**Authors:** Jialu Yao, Songhao Li, Peixian Liang, Xiaowei Xu, David Elder, Zhi Huang

**Affiliations:** 1 Department of Electrical and Systems Engineering, University of Pennsylvania, Philadelphia, PA USA; 2 Department of Pathology and Laboratory Medicine, Perelman School of Medicine, University of Pennsylvania, Philadelphia, PA USA; 3 Department of Biostatistics, Epidemiology and Informatics, Perelman School of Medicine, University of Pennsylvania, Philadelphia, PA USA

**Keywords:** Cancer, Computational biology and bioinformatics, Mathematics and computing

## Abstract

Melanoma is one of the top 5 cancer types, causes most deaths among skin cancers, and can be frequently misdiagnosed. Recent pathology image foundation models remain difficult to make accurate differential diagnosis across over forty melanocytic neoplasm histologic subtypes. Motivated by the diagnostic reasoning process of dermatopathologists, we curated a high-quality image and knowledge corpus database containing 2893 images and 1102 knowledge entries annotated by expert dermatopathologists at the University of Pennsylvania. Leveraging this multi-modal dataset, we present “Melan-Dx”, a knowledge-enhanced AI framework that augments frozen pathology vision-language models through retrieval from a curated vision-knowledge database, improving differential diagnosis at both patch and whole-slide levels. Melan-Dx, at its best performance, demonstrates 0.869 accuracy for binary classification, 0.699 Top-1 accuracy among forty-class classification, 0.915 ROC AUC for few-shot WSI tasks, and 0.925 AUPRC for fully supervised WSI tasks. Across all experimental settings, Melan-Dx shows improvements up to 13.8% over linear and fully finetuned methods, 23–70.6% over zero-shot approaches and up to 8.4% improvements in whole slide image classification. These findings suggest that a query database with a knowledge-enhanced AI framework can further improve existing pathology foundation models without fine-tuning the vision backbone. The code is publicly available at https://www.github.com/zhihuanglab/Melan-Dx-code.

## Introduction

Skin cancer is one of the most common cancers in the world^1^. Among all types, melanocytic tumors cause the majority of skin cancer-related deaths. In 2024 alone, it was estimated that 100,640 new cases of melanoma would be diagnosed and more than 8290 people would die from this disease^2^. Melanoma is also one of the top five cancers for both men and women^2^. Early and accurate diagnosis of melanocytic neoplasms is critical because treating melanoma in its early stages can significantly improve patient outcomes^3^.

The current gold standard for diagnosis is histopathological examination, where pathologists carefully examine tissue slides under the microscope^4^. Despite its importance, misdiagnosis, including both under- and over-diagnosis, remains common^5^, partly due to the high similarity among different subtypes of melanocytic neoplasms and the subjectivity of visual interpretation. A recent study^6^ showed that an estimated 49.7% of melanomas diagnosed in white men and 64.6% in white women were overdiagnosed in 2018. Early detection and treatment of melanoma significantly improve patient prognosis and survival outcomes^3^.

Even among experienced pathologists, there is substantial disagreement when diagnosing moderately atypical lesions and early melanomas. This diagnostic uncertainty has serious consequences: underdiagnosis leads to delayed treatment and disease progression^7^, while overdiagnosis results in unnecessary anxiety, invasive procedures, and wasted healthcare resources^8^. These diagnostic challenges are particularly problematic in areas with limited access to expert dermatopathologists. Current solutions like seeking second opinions or consulting specialists are time-consuming, expensive, and not always available. Recent studies emphasize that successful clinical AI in dermatology relies on safe deployment, fairness, and effective human-AI collaboration. Close cooperation between clinicians and AI systems is essential to improve reliability and clinical impact^9–16^. Therefore, there is an urgent need to improve diagnostic accuracy and reliability through innovative approaches.

Machine learning and deep learning-based algorithm development for pathology images^17–20^, especially skin cancer^21,22^ has attracted growing attention over the past decade, particularly in melanoma identification^23–25^. These endeavors provide promising avenues for automating the diagnosis of highly heterogeneous gigapixel pathology images. However, significant limitations persist as more than 2000 dermatological entities^26^ exist, with many being subtle, and diagnosis does not simply rely on inspecting microscopy images but also on clinical history and other unformatted text information. Recent advances in medical image analysis have explored multi-scale and feature-fusion architectures to enhance representation learning across modalities and spatial contexts^27–30^. To address these multimodal diagnostic requirements, vision-language models (VLMs) have emerged as a powerful solution in medical AI^31–33^. Pathology vision-language foundation models such as PLIP^34^, CONCH^35^, PathGen^36^, and MUSK^37^ combine visual understanding with language-based reasoning, enabling the integration of both image features and textual medical knowledge. While it is recommended using those out-of-box or post-finetuned foundation models for downstream tasks, several limitations remain. First, they are typically trained on large, unstructured datasets and may underperform on specific disease categories like melanocytic tumors. Second, existing foundation models usually rely only on image features and simple text captions without effectively using additional knowledge sources like textbook definitions, diagnostic criteria, or histological patterns. Third, the training of such models often requires large computational resources, making them hard to fine-tune in resource-limited settings. These limitations highlight a need for frameworks that can explicitly incorporate structured medical knowledge and retrieval-based understanding to improve both accuracy and explainability in specialized diagnostic settings.

One of the key challenges in improving AI-based diagnosis for melanocytic neoplasms is the lack of structured, high-quality, multi-modal datasets. Unlike general dermatology datasets (e.g., HAM10000^38^ or PAD-UFES-20^39^), dermatopathology datasets are much smaller and harder to access. Some public databases, such as TCGA^40^ and Quilt-1M^41^, offer large amounts of pathology images, but they are often unstructured or lack accurate labels for subtypes of melanocytic neoplasms.

To address these issues, we propose “Melan-Dx”, an evidence-based vision-knowledge enhancement framework designed specifically for melanocytic neoplasms histopathology. Melan-Dx works on top of any existing pathology VLMs without fine-tuning their vision encoder. Our work has two main contributions. First, we built a structured dermatopathology database, “*Penn Melan-Dx knowledge atlas*”, with over 2800 high-quality examples across 44 melanocytic disease classes. We also summarize detailed knowledge for each class and organize the data in a hierarchical format following the WHO classification of tumors and the Melanocytic Pathology Assessment Tool and Hierarchy for Diagnosis (MPATH-Dx) histology classification^42^. Second, we use this curated dataset to train a vision-knowledge framework that enhances both image and knowledge features through knowledge-enhanced contrastive learning. The framework includes two fusion modules: one for combining input images with reference images (Melan-Dx database), and another for integrating knowledge into the final prediction. We design both local and global contrastive losses to help the model learn strong and explainable representations. Our experimental results show that Melan-Dx improves performance across various benchmarks compared to baseline models on patch-level tasks. Additionally, Melan-Dx achieves dramatic training time reductions at the patch level compared to fully finetuned foundation models while maintaining superior performance. In addition, we also evaluate Melan-Dx on whole slide image (WSI) datasets. We conduct both few-shot and complete training experiments, which demonstrate that our method improves diagnostic performance across a range of training scenarios.

In summary, this work presents a complete pipeline for building an evidence-based AI diagnosis system for melanocytic neoplasms. We construct a large, structured dermatopathology atlas for melanocytic neoplasms, including high-quality image-text pairs and expert-curated medical knowledge. We propose a novel vision-knowledge enhancement framework that integrates image and knowledge features via local and global contrastive learning. We demonstrate that our framework achieves high performance, training efficiency and explainability, with strong generalization across patch-level and WSI-level tasks. Our approach demonstrates the importance of combining structured medical knowledge with visual features to improve AI-based diagnosis in pathology. The system not only provides accurate predictions but also offers transparent reasoning that can help pathologists understand the basis of each diagnosis.

## Results

### Curate high-quality, structured dermatopathology image-text-knowledge dataset

**Fig. 1:**
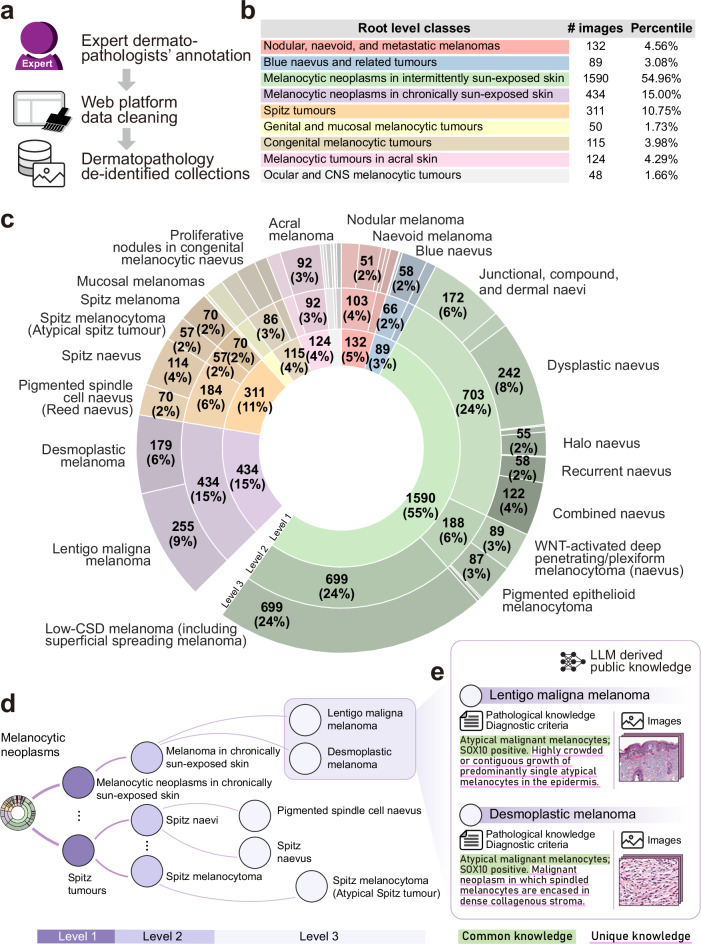
Construction and organization of the Melan-Dx dermatopathology atlas. **a** Dataset construction workflow showing the three-stage curation process. **b** Root-level disease classes with image counts and percentages. **c** Hierarchical organization of melanocytic neoplasm categories following WHO classification. **d** Knowledge graph structure showing relationships between disease categories. **e** Examples of structured knowledge integration with pathological descriptions and diagnostic criteria.

To enable comprehensive evaluation of melanocytic neoplasm diagnosis, we constructed a hierarchically structured dermatopathology database, “*Penn Melan-Dx knowledge atlas*”. This database contains thousands of manually annotated image-text pairs from our private collection (X. Xu and D. Elder) at the University of Pennsylvania (Fig. 1a). Through stringent annotations and post-cleaning by expert dermatopathologists manually via our online collaborative platform (Supplementary Fig. S1), all data were mapped to the WHO classification of tumor for “melanocytic neoplasms” taxonomy. The final *atlas* encompasses 2893 high-quality pathology images spanning 44 distinct melanocytic neoplasm categories. The dataset comprises two primary components: 2699 manually annotated images validated by board-certified dermatopathologists and 194 images sourced from WHO classification materials. The dataset exhibits a hierarchical structure with nine root-level disease classes, ranging from benign nevi to malignant melanomas across different anatomical sites and exposure patterns (Fig. 1b).

The *atlas* follows the WHO hierarchical classification system for melanocytic neoplasms, as visualized in Fig. 1c. This standardized clinical classification enables systematic organization from broad disease categories to specific diagnostic subtypes, supporting both clinical workflow and AI model training.

Within this three-tier structure, the largest category, “Melanocytic neoplasms in intermittently sun-exposed skin”, dominates the dataset with 1590 images (55%) (Supplementary Fig. S2). This category further subdivides into multiple subcategories, being “low-CSD melanoma, including superficial spreading melanoma” (699 images, 24%) and “dysplastic nevus” (242 images, 8%). Additional subcategories include “junctional, compound, and dermal naevi” (172 images, 6%), “combined nevus” (122 images, 4%), “WNT-activated deep penetrating/plexiform melanocytoma” (89 images, 3%), “pigmented epithelioid melanocytoma” (87 images, 3%) and so on.

The second major category, “Melanocytic neoplasms in chronically sun-exposed skin” (434 images, 15%), encompasses lesions typically found in areas of chronic photoaging. This category primarily includes “lentigo maligna melanoma” (255 images, 9%) and “desmoplastic melanoma” (179 images, 6%). And “Spitz tumors” represent a significant diagnostic challenge with 311 images (11%), subdivided into “Spitz nevus” (114 images, 4%), “Spitz melanocytoma” or “atypical Spitz tumor” (57 images, 2%), “Spitz melanoma” (70 images, 2%), and “pigmented spindle cell nevus or Reed nevus” (70 images, 2%).

To enhance the interpretability and clinical utility of our *atlas*, we integrated comprehensive medical knowledge for each disease category (Fig. 1d). The knowledge graph structure demonstrates the hierarchical relationships between different melanocytic neoplasm categories, following the WHO classification system. In our final de-identified collection, each image is a detailed region of interest snapshot at either low-power or high-power, manually matched to a specific disease category by two experienced dermatopathologists (X.X. and D.E.), and has a well-described text caption. In addition, each disease category is enriched with knowledge summaries and characteristic descriptions, which are summarized by a large language model (LLM) from publicly available medical resources (Fig. 1e). Each disease category contains structured knowledge entries including pathological descriptions and diagnostic criteria. Different diseases may have common and unique descriptions. For example, lentigo maligna melanoma is characterized by “*atypical malignant melanocytes; SOX10 positive. Highly crowded or contiguous growth of predominantly single atypical melanocytes in the epidermis*”, while desmoplastic melanoma is described as “*atypical malignant melanocytes; SOX10 positive. Malignant neoplasm in which spindled melanocytes are encased in dense collagenous stroma*”.

### Developing a knowledge-enhanced vision-language framework for melanocytic neoplasms

**Fig. 2:**
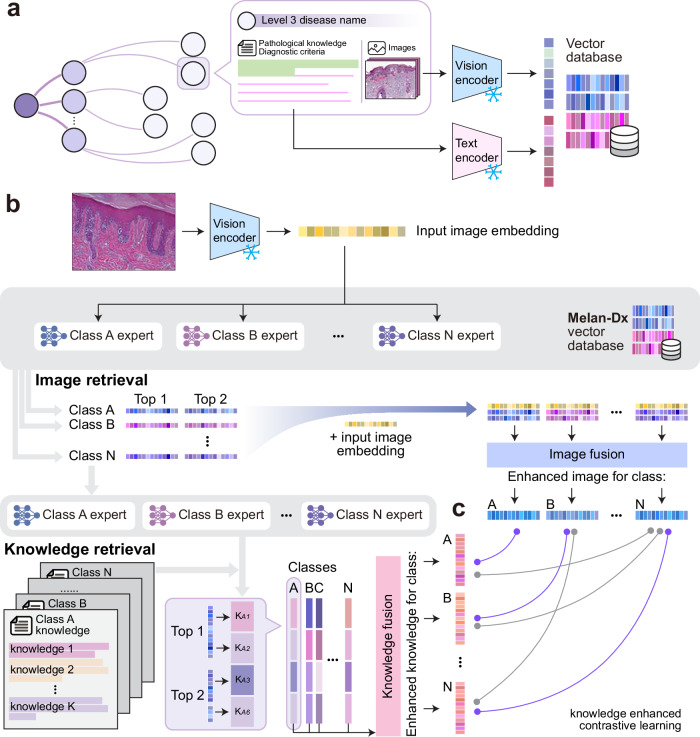
Overview of the Melan-Dx knowledge-enhanced vision-language framework architecture. **a** Construction of the multimodal vector database containing structured pathological knowledge, diagnostic criteria, and expert-validated pathology images for each disease category. Both visual and textual modalities are encoded and stored for further training. **b** Dual-arm architecture showing the image arm (top) and knowledge arm (bottom). The image arm performs class-specific visual retrieval using expert modules to identify relevant exemplars, followed by fusion block to generate enhanced image representations (Top 1 and Top 2 denote the first and second highest weighted retrieved images). The knowledge arm performs cross-modal retrieval to incorporate relevant medical knowledge for each candidate class (*K*_*A*1_, *K*_*A*2_, etc. indicate the retrieved knowledge entries corresponding to class A). **c** Knowledge-enhanced contrastive learning strategy that aligns enhanced image and knowledge representations.

To leverage the structured dermatopathology *atlas* for better melanocytic neoplasm diagnosis, we developed Melan-Dx, a knowledge-enhanced vision-language framework that integrates visual features with domain-specific medical knowledge through a dual-arm architecture (Fig. 2). It consists of an image arm and a knowledge arm to process visual and textual information simultaneously. Unlike conventional VLMs^34–37^ that rely solely on paired image-text training, our approach explicitly incorporates structured medical knowledge through class-specific expert modules and retrieval-augmentation.

Before training the model, we build a vector database (Fig. 2a) that stores both visual and textual embeddings from *Penn Melan-Dx knowledge atlas*. For every disease category in our hierarchical *atlas*, we include the following: (i) manually verified pathological knowledge and diagnostic criteria; and (ii) representative pathology images verified by board-certified dermatopathologists. We then encode the text with a pretrained language encoder and the images with a pretrained vision encoder, and save the resulting dense vectors for retrieval during training and inference.

The model takes a query pathology image as input and processes it through both arms to generate enhanced representations that capture both visual morphological features and semantic medical knowledge. The architecture is designed to mimic the diagnostic reasoning process of dermatopathologists, who combine visual pattern recognition with extensive domain knowledge when making differential diagnoses.

#### Image arm: visual retrieval and fusion

The image arm performs class-specific visual retrieval and fusion to enhance query image representations (Fig. 2b). Given a query image, we first extract its feature embedding using a pre-trained vision encoder. The system then uses class-specific expert modules to find the most relevant visual examples from our *atlas*. For each candidate disease class, an expert module computes similarity weights between the query image and stored reference images. The retrieved image embeddings are then processed through a fusion block to integrate relevant visual information. Finally, the enhanced image embeddings are aggregated by weighted summation to produce a single enhanced image representation for each class.

#### Knowledge arm: cross-modal knowledge retrieval

In parallel, the knowledge arm performs cross-modal retrieval to incorporate relevant medical knowledge (Fig. 2b). Each disease class is associated with structured knowledge entries containing histological features, diagnostic criteria, and differential diagnoses. Similar to the image arm, class-specific experts compare the query image embedding with stored knowledge embeddings and compute attention scores to identify the most relevant medical knowledge for each class. The retrieved knowledge embeddings are processed through the fusion block to generate enhanced knowledge representations.

#### Associating image and diagnosis with knowledge-enhanced contrastive learning

Melan-Dx uses a knowledge-enhanced contrastive learning strategy to align visual and textual representations (Fig. 2c). Our approach implements two types of supervision. First, we use local contrastive loss to ensure precise matching between enhanced image and knowledge embeddings within each class (see “Methods” for detail). This helps the model learn class-specific patterns and characteristics. Second, we apply global contrastive loss to promote instance-level discriminability across the entire batch, encouraging the model to distinguish between different samples and different classes. This dual-supervision strategy allows the model to capture both class-specific diagnostic features and instance-level distinctions, making it well-suited for complex melanocytic neoplasm differential diagnosis.

### Melan-Dx improves patch-level differential diagnosis

To evaluate the effectiveness of our Melan-Dx framework, we conducted comprehensive experiments comparing our knowledge-enhanced approach against state-of-the-art vision-language foundation models. Since no other publicly available vision-language dataset exists for melanocytic neoplasms, we performed the evaluation on our internal test split. A total of 60 performance comparisons were conducted (4 foundation models × 3 baseline methods × 5 evaluation tasks and metrics). Melan-Dx demonstrated statistically significant improvements over baseline approaches in nearly all cases. For the remaining 4 comparisons where Melan-Dx did not achieve significant superiority, it maintained equivalent performance levels as confirmed by paired Student’s *t* tests.

We excluded disease categories with fewer than 3 images and focused on 40 melanocytic neoplasm classes for multi-class evaluation. Additionally, we conducted binary classification by grouping diseases into melanoma versus nonmelanoma categories according to MPATH-Dx assessment criteria. We compared four different approaches across four foundation model backbones (PLIP, PathGen, CONCH, and MUSK): zero-shot classification, linear finetuned foundation models (trained with a linear classifier on top of frozen foundation models), fully finetuned foundation models, and our Melan-Dx knowledge-enhanced framework. Note that Melan-Dx uses off-the-shelf vision transformers from existing pathology foundation models without any fine-tuning.

**Fig. 3:**
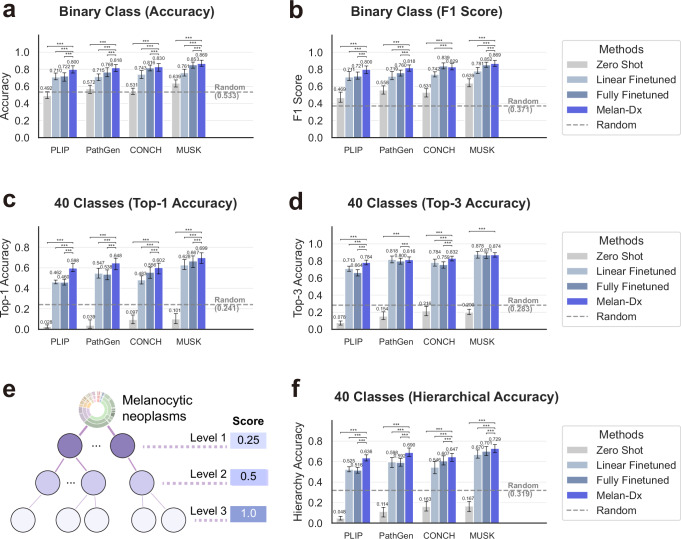
Patch-level classification performance of Melan-Dx across different foundation model backbones and evaluation scenarios. **a** Binary classification weighted F1 scores across four foundation model backbones (PLIP, PathGen, CONCH, MUSK). **b** Binary classification accuracy results across all foundation model backbones. **c** Multi-class Top-1 accuracy on 40 melanocytic neoplasm categories. **d** Multi-class Top-3 accuracy on 40 melanocytic neoplasm categories. **e** Hierarchical scoring scheme based on WHO classification structure. **f** Hierarchical accuracy results across foundation models. Error bars represent 95% confidence intervals from bootstrap resampling. Statistical significance was assessed using paired two-sided Student’s *t* test: *p <0.05, **p <0.01, ***p <0.005. Random baseline represents majority class prediction performance.

For the binary classification task (melanoma vs. nonmelanoma), Melan-Dx consistently outperformed all baseline approaches across nearly all foundation model backbones (Fig. 3a, b). The random baseline achieved an accuracy of 0.533 and an F1 score of 0.371. In terms of accuracy, Melan-Dx achieved substantial improvements: PLIP+Melan-Dx reached 0.800 compared to 0.722 (*P* = 2.81 × 10^−28^) for fully finetuned, 0.710 (*P* = 6.46 × 10^−35^) for linear probing, and 0.492 (*P* = 2.83 × 10^−83^) for zero-shot; PathGen+Melan-Dx achieved 0.818 versus 0.768 (*P* = 6.43 × 10^−19^), 0.715 (*P* = 3.14 × 10^−45^), and 0.572 (*P* = 4.25 × 10^−75^) respectively; CONCH+Melan-Dx attained 0.830 compared to 0.816 (*P* = 4.50 × 10^−4^), 0.743 (*P* = 1.37 × 10^−35^), and 0.531 (*P* = 4.91 × 10^−83^); and MUSK+Melan-Dx reached the highest performance at 0.869 versus 0.853 (*P* = 2.32 × 10^−4^), 0.761 (*P* = 8.2 × 10^−48^), and 0.639 (*P* = 4.08 × 10^−77^) respectively. For F1 scores (Fig. 3b), Melan-Dx showed consistent improvements across PLIP, PathGen, and MUSK backbones. MUSK+Melan-Dx reached the highest performance at 0.869. Notably, CONCH+Melan-Dx achieved 0.829 compared to 0.839 (*P* = 9.78 × 10^−2^) for fully finetuned, showing comparable performance with no statistically significant difference, while still substantially outperforming linear probing (0.742, *P* = 9.22 × 10^−43^) and zero-shot (0.531, *P* = 7.11 × 10^−86^) approaches.

The advantages of Melan-Dx became even more apparent in the challenging 40-class fine-grained classification scenario. The random baseline achieved only 0.241 Top-1 accuracy and 0.283 Top-3 accuracy, respectively. For Top-1 accuracy—an extremely difficult task (Fig. 3c), our approach significantly outperformed all baseline methods across all foundation models: PLIP+Melan-Dx achieved 0.598 compared to 0.460 (*P* = 3.32 × 10^−11^) for fully finetuned, 0.462 (*P* = 1.51 × 10^−35^) for linear finetuned, and 0.028 (*P* =4.99 × 10^−100^) for zero-shot; PathGen+Melan-Dx reached 0.648 versus 0.538 (*P* = 1.32 × 10^−38^), 0.547 (*P* = 9.92 × 10^−34^), and 0.039 (*P* = 7.58 × 10^−117^); CONCH+Melan-Dx attained 0.602 compared to 0.556 (*P* = 3.32 × 10^−11^), 0.483 (*P* = 1.51 × 10^−35^), and 0.097 (*P* = 4.99 × 10^−100^); and MUSK+Melan-Dx achieved the highest performance at 0.699 versus 0.667 (*P* =1.00 × 10^−6^), 0.628 (*P* = 9.31 × 10^−23^), and 0.101 (*P* = 4.57 × 10^−111^), respectively.

The Top-3 accuracy results (where the ground truth falls within the top three predicted classes) (Fig. 3d) further confirmed the benefit of knowledge integration: PLIP+Melan-Dx achieved 0.784 compared to 0.664 (*P* = 1.78 × 10^−45^) for fully finetuned, 0.713 (*P* = 3.24 × 10^−29^) for linear finetuned, and 0.078 (*P*=6.36 × 10^−136^) for zero-shot; PathGen+Melan-Dx reached 0.816 versus 0.800 (*P* = 8.91 × 10^−4^), 0.818 (*P* = 4.84 × 10^−1^), and 0.154 (*P* = 2.38 × 10^−122^); CONCH+Melan-Dx attained 0.832 compared to 0.759 (*P* = 1.34 × 10^−30^) for fully finetuned, 0.784 (*P* = 8.19 × 10^−21^) for linear finetuned, and 0.216 (*P* = 2.18 × 10^−119^) for zero-shot; and MUSK+Melan-Dx achieved 0.874 versus 0.871 (*P* = 2.15 × 10^−1^), 0.878 (*P* = 8.78 × 10^−2^), and 0.200 (*P* = 4.71 × 10^−132^) respectively. CONCH+Melan-Dx and MUSK+Melan-Dx performed comparably to their linear finetuned counterparts with no statistically significant differences. Zero-shot performance was consistently poor across all foundation models, which reflects the difficulty of fine-grained melanocytic neoplasm classification without knowledge enhancement.

### Melan-Dx improves taxonomy understanding of melanocytic neoplasms

To assess the clinical utility of our approach, we evaluated hierarchical accuracy using a structure-aware metric that assigns partial credit based on the WHO classification hierarchy (Fig. 3e, f). This metric is particularly relevant for melanocytic neoplasm diagnosis, where misclassifications within the same diagnostic family (e.g., different types of nevi) are less clinically significant than misclassifications across major categories (e.g., confusing melanoma with benign lesions).

The hierarchical scoring system (Fig. 3e) assigns different weights to classification errors based on their position in the WHO taxonomy: Level 1 errors (broadest categories) receive 0.25 partial credit, Level 2 errors receive 0.5 partial credit, and Level 3 errors (exact matches) receive full 1.0 credit. This approach better reflects real-world clinical consequences where diagnostic errors at higher taxonomic levels carry greater clinical risks. This makes hierarchical accuracy more clinically relevant than Top-3 accuracy, which treats all errors equally.

The hierarchical accuracy results (Fig. 3f) demonstrated consistent improvements with Melan-Dx across all foundation models: PLIP+Melan-Dx achieved 0.636 compared to 0.516 (*P* = 8.13 × 10^−42^) for fully finetuned, 0.525 (*P* = 7.63 × 10^−38^) for linear finetuned, and 0.048 (*P* = 4.07 × 10^−116^) for zero-shot; PathGen+Melan-Dx reached 0.690 versus 0.592 (*P* = 1.94 × 10^−33^), 0.598 (*P* = 1.58 × 10^−36^), and 0.114 (*P* = 3.01 × 10^−121^); CONCH+Melan-Dx attained 0.647 compared to 0.607 (*P* = 1.66 × 10^−12^), 0.546 (*P* = 3.70 × 10^−40^), and 0.163 (*P* = 7.34 × 10^−108^); and MUSK+Melan-Dx achieved the highest performance at 0.729 versus 0.701 (*P* = 4.16 × 10^−11^), 0.670 (*P* = 2.22 × 10^−24^), and 0.167 (*P* = 1.14 × 10^−117^), respectively. The random baseline achieved 0.319 hierarchical accuracy.

### Melan-Dx achieves superior training efficiency

Given that Melan-Dx demonstrated the best overall performance followed by fully finetuned foundation models in our previous experiments, we further compared their training efficiency to assess the practical advantages of our approach. We recorded the training time over 30 epochs for both 40-class and binary classification tasks across all foundation model backbones.

**Fig. 4:**
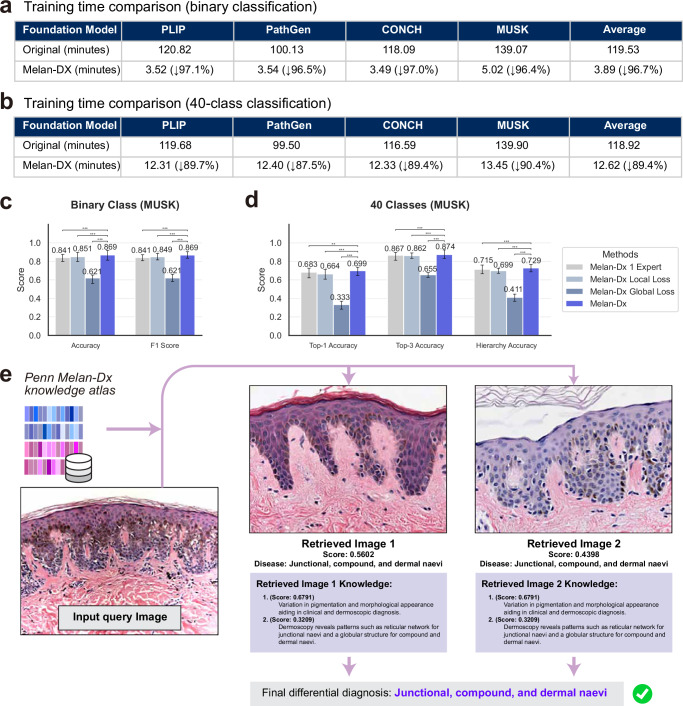
Training efficiency, ablation study, and explainability analysis of Melan-Dx. **a** Training time comparison for binary classification over 30 epochs. **b** Training time comparison for 40-class classification tasks. **c** Ablation study results on binary classification using MUSK backbone. **d** Ablation study results on 40-class classification. Error bars represent 95% confidence intervals from bootstrap resampling. Statistical significance was assessed using two-sided paired Student’s *t* test: *p <0.05, **p <0.01, ***p <0.005. **e** An example shows retrieved images and relevant knowledge with confidence scores for diagnostic transparency.

Benefiting from the knowledge-enhanced architecture, Melan-Dx does not need to fine-tune the vision encoder, therefore is highly efficient to adapt on an established *atlas* (*Penn Melan-Dx knowledge atlas*). For the binary classification task (Fig. 4a), Melan-Dx achieved significant improvements in training efficiency across all foundation models. The original fully finetuned approaches required much more training time: PLIP (120.82 min), PathGen (100.13 min), CONCH (118.09 min), and MUSK (139.07 min), with an average of 119.53 min. In contrast, Melan-Dx substantially reduced training time to: PLIP+Melan-Dx (3.52 min, 97.1% reduction), PathGen+Melan-Dx (3.54 min, 96.5% reduction), CONCH+Melan-Dx (3.49 min, 97.0% reduction), and MUSK+Melan-Dx (5.02 min, 96.4% reduction), which achieves an average training time of only 3.89 min with an overall 96.7% time reduction.

Similar efficiency gains were observed for the challenging 40-class classification task (Fig. 4b). Original training times were: PLIP (119.68 min), PathGen (99.50 min), CONCH (116.59 min), and MUSK (139.90 min), averaging 118.92 min. Melan-Dx reduced these to: PLIP+Melan-Dx (12.31 min, 89.7% reduction), PathGen+Melan-Dx (12.40 min, 87.5% reduction), CONCH+Melan-Dx (12.33 min, 89.4% reduction), and MUSK+Melan-Dx (13.45 min, 90.4% reduction), which reaches an average of 12.62 min with a 89.4% time reduction. These results demonstrate that Melan-Dx not only achieves superior diagnostic performance but also provides substantial computational efficiency, making it highly practical for real-world deployment.

### Each component of Melan-Dx contributes to overall effectiveness

Since MUSK+Melan-Dx demonstrated the strongest overall performance across all previous comparisons, we conducted ablation studies using this model to exploit the contribution of different components. We evaluated the same metrics as in Fig. 3 to ensure consistent comparison. We compared four configurations: Melan-Dx with same expert for all classes (Melan-Dx 1 Expert), Melan-Dx with only local contrastive loss (Melan-Dx Local Loss), Melan-Dx with only global contrastive loss (Melan-Dx Global Loss), and the complete Melan-Dx framework with multi experts, local and global loss (Melan-Dx).

For binary classification (Fig. 4c), complete Melan-Dx showed 0.869 accuracy, while ablated versions reduce the performance: 1 expert (0.841, *P* = 3.00 × 10^−10^), local loss only (0.851, *P* = 1.58 × 10^−7^), and global loss only (0.621, *P* = 4.55 × 10^−77^). F1 score exhibits similar patterns: 0.869 for complete framework versus 0.841 (*P* = 2.20 × 10^−11^), 0.849 (*P* = 9.36 × 10^−8^), and 0.621 (*P* = 1.79 × 10^−72^), respectively. For Top-1 accuracy in 40-class classification (Fig. 4d), the complete Melan-Dx achieved 0.699, while ablated versions showed reduced performance: 1 expert (0.683, *P* = 5.45 × 10^−3^), local loss only (0.664, *P* = 1.93 × 10^−11^), and global loss only (0.333, *P* = 1.44 × 10^−83^). Top-3 accuracy followed similar patterns with complete Melan-Dx at 0.874 compared to 0.867 (*P* = 4.41 × 10^−3^), 0.862 (*P* = 3.03 × 10^−4^), and 0.655 (*P* = 1.19 × 10^−74^), respectively. Hierarchical accuracy demonstrated the same trend: complete Melan-Dx 0.729 versus 0.715 (*P* = 1.97 × 10^−3^), 0.699 (*P* = 4.30 × 10^−11^), and 0.411 (*P* = 1.22 × 10^−82^) for the ablated configurations. Across all evaluation scenarios, removing any component of Melan-Dx resulted in statistically significant performance degradation as confirmed by paired Student’s t-tests, which demonstrates each component is essential for optimal overall framework performance.

### Melan-Dx provides evidence-based diagnosis

To show how Melan-Dx works, we present an example of its retrieval process (Fig. 4e). For a given input image, the system finds the most similar images and relevant medical knowledge from our *atlas*. Melan-Dx image arm retrieved two relevant reference images in class “Junctional, compound, and dermal naevi” with scores of 0.5602 and 0.4398. These images show similar tissue patterns to the input image. The knowledge arm also retrieved relevant medical knowledge with confidence scores. The top knowledge entry (score: 0.6791) described “Variation in pigmentation and morphological appearance aiding in clinical and dermoscopic diagnosis.” The second entry (score: 0.3209) provided specific details: “Dermoscopy reveals patterns such as reticular network for junctional naevi and a globular structure for compound and dermal naevi.” This example shows how Melan-Dx combines visual similarities with medical knowledge to support diagnosis. The scores can help doctors understand how confident the system is about each piece of evidence, making the diagnostic process clear and reliable.

### Melan-Dx helps enhance few-shot whole slide image classification

To evaluate the scalability and clinical applicability of our Melan-Dx framework, we extended our analysis to WSI classification using the HISTAI skin dataset^43^. This dataset provides case-specific diagnostic results, which we filtered using GPT-4o to match only cases with clear melanoma and nonmelanoma diagnoses. After two rounds of strict matching, we retained consistently matched cases and manually verified the diagnostic results. We randomly selected 1000 WSIs from this filtered dataset for the experiment.

**Fig. 5:**
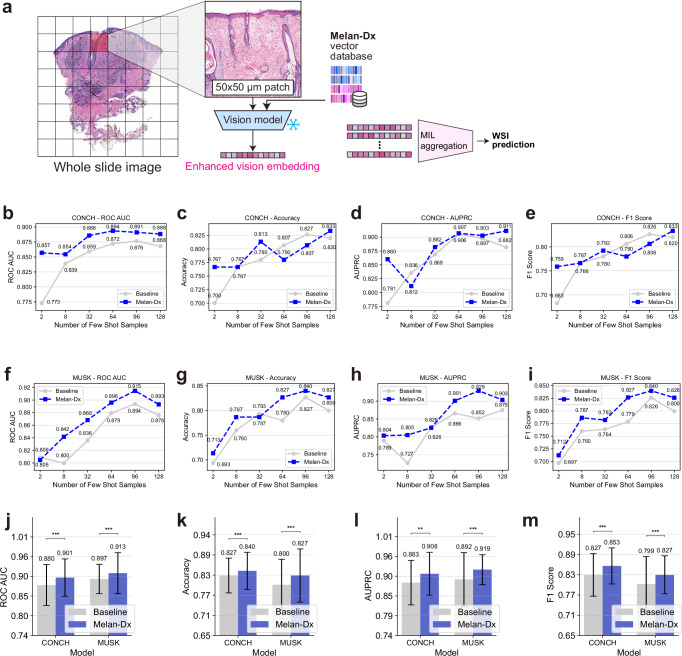
Whole slide image classification performance using Melan-Dx on the HISTAI skin dataset. **a** WSI classification pipeline with patch extraction, Melan-Dx enhancement, and multiple instance learning (MIL) aggregation for slide-level prediction. **b–e** Few-shot learning results using CONCH backbone across different sample sizes (2-128 shots) for ROC AUC, accuracy, AUPRC, and F1 score. **f–i** Few-shot learning results using MUSK backbone with the same metrics and sample sizes as (**b–e**). **j–m** Supervised learning performance comparison for both CONCH and MUSK backbones. Error bars represent 95% confidence intervals from bootstrap resampling. Statistical significance was assessed using two-sided paired Student’s *t* test: *p < 0.05, **p < 0.01, ***p < 0.005.

For Melan-Dx WSI classification pipeline (Fig. 5a), each WSI is divided into 512 × 512 pixel patches at ×10 magnification, which are processed through our Melan-Dx-enhanced framework to generate enhanced vision embeddings. These patch-level embeddings are subsequently aggregated using attention-based multiple instance learning (ABMIL)^44^ to make slide-level predictions. Since Melan-Dx with CONCH and MUSK backbones demonstrated superior performance in previous experiments, we focused our WSI evaluation on these two configurations, comparing them against their corresponding baseline foundation models. We totally conducted 48 few-shot comparisons (2 foundation models × 4 metrics × 6 shot settings) and 8 supervised learning comparisons (2 foundation models × 4 metrics), and Melan-Dx outperforms baseline models in most cases.

We conducted comprehensive few-shot learning experiments using varying numbers of training samples (2, 8, 32, 64, 96, and 128 shots) across four evaluation metrics (ROC AUC, accuracy, AUPRC, and F1 score) to assess performance. For Melan-Dx with CONCH vision backbone (Fig. 5b–e), CONCH+Melan-Dx improved foundation model performance in most scenarios, while maintaining similar performance in the remaining cases. Particularly notable were the substantial improvements at 2 shots across all four metrics: ROC AUC (0.857 vs. 0.773), accuracy (0.767 vs. 0.700), AUPRC (0.860 vs. 0.781), and F1 score (0.759 vs. 0.683). For 64 and 96 shots, accuracy and F1 score showed some degradation, but ROC AUC and AUPRC performance showed improvement. For ROC AUC, we achieved remarkable performance of 0.894 at 64 shots compared to 0.872 for baseline. For accuracy, AUPRC, and F1 score, we reached peak performance at 128 shots with improvements over CONCH baseline: accuracy (0.833 vs. 0.820), AUPRC (0.911 vs. 0.882), and F1 score (0.833 vs. 0.820).

For Melan-Dx with MUSK vision backbone (Fig. 5f–i), MUSK+Melan-Dx demonstrated improvements in nearly all scenarios, with only three cases showing equivalent or similar performance levels. Remarkably, at 96 shots, all four metrics reached their peak performance: ROC AUC (0.915 vs. 0.894 baseline), accuracy (0.840 vs. 0.827 baseline), AUPRC (0.929 vs. 0.852 baseline), and F1 score (0.840 vs. 0.826 baseline). Our Melan-Dx few-shot performance was even higher than MUSK model supervised learning performance, which highlights the effectiveness of our knowledge enhancement method in few-shot learning scenarios for WSI classification tasks.

### Melan-Dx helps enhance fully supervised whole slide image classification

Under fully supervised learning setting with ABMIL aggregation (Fig. 5j–m), Melan-Dx also demonstrated significant improvements over baseline foundation models as confirmed by paired Student’s *t* tests. For CONCH, Melan-Dx achieved superior performance: ROC AUC (0.897 vs. 0.883, *P* = 9.35 × 10^−7^), accuracy (0.820 vs. 0.800, *P* = 1.54 × 10^−4^), and F1 score (0.820 vs. 0.799, *P* = 1.28 × 10^−3^). For AUPRC, CONCH+Melan-Dx showed comparable performance (0.894 vs. 0.898, *P* = 6.12 × 10^−2^) with no statistically significant difference. For MUSK, consistent improvements were observed across all metrics: ROC AUC (0.908 vs. 0.870, *P* = 3.15 × 10^−9^), accuracy (0.827 vs. 0.780, *P* = 6.24 × 10^−6^), AUPRC (0.920 vs. 0.838, *P* =6.20 × 10^−17^), and F1 score (0.827 vs. 0.777, *P* = 2.94 × 10^−10^).

To further validate the effectiveness of our knowledge-enhanced approach on WSI-level tasks, we compared Melan-Dx against TITAN^45^, a recently published WSI-level foundation model, on the HISTAI skin dataset. Unlike patch-level foundation models, TITAN operates at the WSI level by aggregating patch embeddings into slide-level representations through its specialized aggregation architecture. This architectural difference prevents direct application of Melan-Dx to TITAN’s aggregation module, as our framework is designed to enhance individual patch representations before aggregation. However, TITAN relies on patch-level vision-language foundation models as its feature extractor to generate initial patch embeddings, which are subsequently aggregated into slide-level features. This dependency provides an opportunity to evaluate whether enhancing the patch-level backbone with Melan-Dx can improve the overall WSI-level classification performance when combined with TITAN’s aggregation method.

TITAN requires CONCH v1.5 as its patch-level feature extractor for slide-level embedding aggregation. However, since CONCH v1.5 does not publicly release its text encoder, we cannot apply Melan-Dx enhancement to this backbone. Therefore, we conducted comparisons using two patch-level encoders (CONCH v1.0 and MUSK) that have publicly available vision and text encoders, which enable Melan-Dx enhancement. For each backbone, we evaluated four configurations: (1) baseline patch features aggregated with ABMIL (Baseline + ABMIL), (2) Melan-Dx enhanced patch features aggregated with ABMIL (Melan-Dx + ABMIL), (3) baseline patch features aggregated with TITAN’s method (Baseline + TITAN), and (4) Melan-Dx enhanced patch features aggregated with TITAN’s method (Melan-Dx + TITAN). This design allows us to assess whether Melan-Dx enhancement at the patch level can improve the WSI-level performance of TITAN across different backbones (CONCH and MUSK), and whether the benefits of patch-level knowledge enhancement are consistent regardless of the subsequent aggregation strategy (ABMIL and TITAN).

Supplementary Fig. S5 presents the experimental results. For TITAN aggregation, we added a linear projection layer before the TITAN encoder to align the patch embedding dimensions, and a linear classification layer after the encoder for final predictions. During training, we optimized the TITAN encoder along with these two linear layers. Using TITAN’s advanced aggregation method, we observed substantial improvements with Melan-Dx enhancement. For the CONCH v1.0 backbone, Baseline + TITAN achieved 0.886 ROC AUC, 0.813 accuracy, 0.891 AUPRC, and 0.813 F1 score. Melan-Dx + TITAN significantly improved all metrics to 0.910 ROC AUC (*P* = 2.24 × 10^−4^), 0.860 accuracy (*P* = 4.77 × 10^−14^), 0.911 AUPRC (*P* = 9.32 × 10^−3^), and 0.860 F1 score (*P* = 4.59 × 10^−11^).

For the MUSK backbone with TITAN aggregation, the baseline achieved strong performance with 0.916 ROC AUC, 0.807 accuracy, 0.917 AUPRC, and 0.806 F1 score. Melan-Dx + TITAN reached 0.840 accuracy (*P* = 4.82 × 10^−8^), 0.925 AUPRC (*P* = 8.60 × 10^−3^), and 0.840 F1 score (*P* = 1.90 × 10^−7^), representing significant improvements of 4.1, 0.9, and 4.2% relative gains, respectively. The ROC AUC was 0.914, slightly lower than baseline but not statistically significant (*P* = 8.50 × 10^−1^).

For completeness, we also evaluated TITAN with its original patch encoder CONCH v1.5, although Melan-Dx enhancement cannot be applied due to the unavailability of CONCH v1.5’s text encoder. As shown in Supplementary Fig. S5e, TITAN with CONCH v1.5 achieved 0.917 ROC AUC, 0.834 accuracy, 0.924 AUPRC, and 0.834 F1 score. Notably, while TITAN was extensively pre-trained with CONCH v1.5 (does not release its text encoder), our Melan-Dx enhanced approaches using publicly available patch-level encoders (CONCH v1.0 and MUSK) achieved comparable or even superior performance on certain metrics. These results show Melan-Dx can effectively scale to WSI analysis and demonstrate excellent performance for potential clinical adoptions and applications.

## Discussion

In this study, we present Melan-Dx, a knowledge-enhanced vision-language framework specifically designed for melanocytic differential diagnosis. Our approach addresses a critical clinical challenge in dermatopathology, where accurate differentiation among over 40 melanocytic neoplasm subtypes is very difficult even for experienced pathologists.

Our structured dermatopathology *atlas* provides a significant advance for specialized medical AI. Unlike general pathology vision-language datasets, our *atlas* offers comprehensive melanocytic neoplasm coverage with expert-validated annotations. We organize the data following the WHO classification standards. This addresses a key gap where previous datasets lacked diversity in melanocytic lesions or detailed knowledge for precise diagnosis. Our approach shows the importance of adding structured medical knowledge to VLMs. Foundation models are powerful but struggle with the subtle differences in melanocytic diagnosis. By combining retrieval-based learning with class-specific expert modules, Melan-Dx bridges the gap between general computer vision and specialized diagnostic expertise.

Each component in the Melan-Dx framework contributes uniquely to the model’s overall performance. The class-specific expert modules allow the model to adapt to diverse morphological patterns across disease categories, improving its ability to handle fine-grained diagnostic variations. The image and knowledge fusion modules help the model combine the retrieved information and reason jointly using both visual and knowledge cues. In addition, the local and global contrastive losses ensure that the model learns both fine-level intra-class consistency and broader inter-class separation. Our ablation results further confirm that each component, including class-specific experts, fusion modules, and contrastive losses, contributes critically to the overall framework performance. The explainability of Melan-Dx reveals that the model learns meaningful visual patterns, which are consistent with the hierarchical taxonomy defined in the WHO classification of melanocytic neoplasms. Melan-Dx demonstrated excellent performance across multiple evaluation scenarios. For patch-level classification, MUSK+Melan-Dx achieved the highest Top-1 accuracy of 0.699 in 40-class classification and 0.869 accuracy in binary classification, significantly outperforming all baseline approaches. Beyond superior accuracy, our framework provides substantial training efficiency advantages. Compared to fully finetuned foundation models, we achieved an average 96.7% time reduction for binary classification and 89.4% time reduction for 40-class classification across all four foundation models. Additionally, Melan-Dx provides transparent diagnostic evidence through its retrieval mechanism. It shows similar pathological images and relevant medical knowledge with confidence scores for each diagnosis, enabling clinicians to understand the reasoning behind predictions. For WSI classification, our framework also achieved remarkable results. MUSK+Melan-Dx reaches 0.915 ROC AUC in few-shot scenarios with ABMIL aggregation and achieves 0.925 AUPRC under the fully supervised setting with TITAN aggregation. These results demonstrate that Melan-Dx achieves superior diagnostic performance using significantly fewer training resources while maintaining clinical explainability. We also performed paired two-sided Student’s t-tests with 95% confidence intervals obtained via bootstrap resampling in the main experiments to assess the statistical reliability of the performance improvements.

Despite these advances, several limitations should be considered. First, our dataset size is limited by available resources. Fine-grained, accurate annotation requires very experienced dermatopathologists with many years of training, which restricts our dataset’s scale. Within melanocytic neoplasms, the model shows strong robustness and generalization, as confirmed on both patch-level and whole-slide image tasks. However, the Melan-Dx framework can be applied to other domains when similar image-knowledge atlases are available. It can be scaled using initial annotations by general pathologists, followed by expert validation of uncertain cases. With curation and knowledge extraction, the framework can expand efficiently and support broader clinical applications. Second, the Melan-Dx framework can be applied to broader medical domains beyond melanocytic neoplasms, but our current work is limited to this specific area due to resource constraints. Third, the model architecture can be further improved. The fusion module structure could be better designed to enhance performance, and more effective training strategies could be explored. Fourth, Melan-Dx relies on pretrained pathology foundation models and a curated multimodal database to provide high-quality visual and textual embeddings. Extending the framework to other domains requires both a suitable pretrained vision-language encoder and a well-structured image knowledge database. Future work will focus on constructing domain-adaptive encoders and expanding large-scale multimodal knowledge bases to enhance the generalizability of Melan-Dx across different medical specialties. Fifth, the current Melan-Dx mainly focuses on vision-language foundation models. However, it can also be extended to image-only models such as H-Optimus-1^46^, UNI-2^31^, or Virchow-2^47^. For these models, the vision encoder can be frozen, and a lightweight biomedical text encoder (e.g., BiomedBERT^48^) can be fine-tuned through large-scale contrastive learning on publicly available pathology datasets such as OpenPath^34^. This process enables vision-language alignment for originally image-only architectures, which makes them compatible with the Melan-Dx framework.

Melan-Dx represents a significant advance in AI-assisted melanocytic lesion diagnosis. Our results show that integrating domain-specific knowledge can substantially improve diagnostic performance across multiple scenarios. The framework addresses key limitations of existing approaches and provides a foundation for future specialized medical AI development. Our work demonstrates the importance of domain expertise in medical AI systems and shows the value of structured knowledge resources. While challenges remain in scalability and multi-modal integration, our results establish a clear path for more effective diagnostic pathology tools. We acknowledge that potential biases in the training data and the risk of over-reliance on AI predictions remain important ethical and clinical concerns. Melan-Dx is therefore designed to facilitate human-AI collaboration^49^, assisting pathologists in improving diagnostic consistency and efficiency rather than replacing human expertise. Combined with transparent AI diagnosis and human oversight, this collaboration can enhance trust and safety in clinical decision-making. In future work, we plan to conduct user studies comparing diagnostic performance across expert + AI, expert only, and AI only settings to further investigate this human-AI collaboration. In terms of deployment feasibility, Melan-Dx is lightweight and efficient. On an NVIDIA A100 GPU, the average inference time for 100 ROI samples was about 0.35 seconds using the MUSK backbone. Given its moderate model size, Melan-Dx can also be deployed on standard laptops for routine use, enabling practical integration into clinical workflows. The methods demonstrated in Melan-Dx can serve as a template for developing clinically relevant knowledge-enhanced AI diagnostic tools across various medical specialties.

## Methods

### Construction of Penn Melan-Dx knowledge atlas

To enable fine-grained melanocytic neoplasm diagnosis, we constructed a structured dermatopathology dataset that integrates visual data, expert annotations, and hierarchical disease knowledge. All images were manually annotated by expert dermatopathologists to ensure reliability. Two board-certified dermatopathologists reviewed and labeled 2,699 images using a custom web-based annotation interface (Supplementary Fig. S1). The interface features three panels: a left panel showing raw image folders, a center panel displaying the hierarchical WHO classification taxonomy for melanocytic neoplasms, and a right panel providing visual thumbnails of images assigned to each category. Dermatopathologists could efficiently navigate and reorganize the dataset by dragging images into appropriate disease categories. The interface also supports dynamic taxonomy editing, including adding, renaming, or restructuring classes as needed. During the matching process via the web portal, two expert dermatopathologists carefully mapped the images from their previous folders to the corresponding WHO tumor classification categories. Consensus was reached through mutual verification by inspecting each other’s annotations.

To expand beyond expert annotations, we integrated 194 additional high-quality images from WHO classification materials (These WHO-derived images were used exclusively within the research team and are not included in publicly released embeddings). This resulted in a dataset of 2893 pathology images spanning 44 distinct melanocytic neoplasm categories. The dataset covers a broad spectrum of melanocytic lesions, including benign nevi, intermediate atypical proliferations, and malignant melanomas.

We organized class labels into hierarchical taxonomies based on WHO system for melanocytic neoplasms (Supplementary Fig. S2). This creates a structured framework with nine major diagnostic categories ranging from benign nevi to malignant melanomas. The *atlas* follows a three-tier hierarchical structure that enables systematic categorization from broad anatomical contexts to specific diagnostic entities. Level 1 comprises nine root categories representing distinct anatomical and clinical contexts. “Melanocytic neoplasms in intermittently sun-exposed skin” is the largest category with 1590 images (54.96%), followed by “Melanocytic neoplasms in chronically sun-exposed skin” (434 images; 15.00%) and “Spitz tumors” (311 images; 10.75%). Level 2 subdivides each Level 1 category into 20 intermediate clinical classifications. Level 3 provides the finest diagnostic granularity with 44 specific entities.

To enhance explainability and diagnostic utility, we curated structured domain knowledge associated with each disease class, informed by authoritative pathology references such as the WHO Blue Book. We used GPT-4o as an assistive tool to organize this information into a structured format. Each knowledge entry includes descriptions of histological features, diagnostic criteria, and differential diagnoses. All knowledge summaries were manually reviewed by us. Since the summaries were concise restatements of verified medical definitions rather than new interpretations, after the careful review, no corrections were required, and all outputs were retained as generated. In addition, one of the authors conducted multiple rounds of inspection to verify the accuracy and clarity of each GPT-4o summary. This careful review process ensured that all entries were internally consistent and medically accurate. Also, we performed an additional manual inspection by carefully inspecting the curated knowledge, and confirmed that there is no misleading or incorrect text. We also mapped the 44 disease classes using MPATH-Dx, a widely used melanocytic pathology assessment tool that defines four severity levels (I to IV). This enables hierarchical supervision where disease classes are grouped by severity for downstream classification tasks.

### Model architecture

We designed a dual-arm architecture with an image arm and a knowledge arm to integrate visual features and textual domain knowledge for melanocytic neoplasm diagnosis. The model enhances query image representations by retrieving and fusing relevant visual and knowledge information using class-specific expert modules implemented with multi-head attention. These enhanced representations are aligned using contrastive learning objectives. Both the Image Fusion and Knowledge Fusion modules are implemented as 8-layer Transformers designed to iteratively integrate retrieved information and refine contextual representations. In the image arm, class-specific expert modules first compute attention between the query image and retrieved related images. The resulting embeddings are then fused through the Transformer block to capture inter-image dependencies. In the knowledge arm, a parallel Transformer fusion module refines retrieved knowledge embeddings and models semantic relationships among them. The Transformer architecture was chosen for its strong capacity to model global contextual dependencies and perform adaptive feature integration, which parallels the diagnostic reasoning process of dermatopathologists.

During contrastive learning phase training, the pretrained encoders from the pathology foundation models are kept frozen (i.e., parameters inside the neural networks will not get updated), while the parameters of the image and knowledge arms, including the class-specific expert modules and fusion modules are updated through contrastive learning. The overall training procedure of Melan-Dx is summarized in Algorithm 1, and the inference process is detailed in Algorithm 2. All notations used in this section are summarized in Supplementary Table S1.

#### Image arm: visual retrieval and fusion

Given a query image *I*_*q*_, we first extract its feature embedding **v**_*q*_ using a pre-trained vision encoder (e.g., PLIP or MUSK). To incorporate contextual visual evidence, we perform image-to-image retrieval using a set of class-specific experts, each implemented as a multi-head attention module. These retrieved examples provide additional visual references that help the model refine its predictions by incorporating consistent morphological patterns related to the query image. For each class *c*, an expert ${E}_{c}^{img}$ computes attention weights between the query and candidate support images {**v**_1_, …, **v**_*n*_}, generating a set of class-relevant visual features:

1 $${\widetilde{{\bf{V}}}}_{q}=\{{{\bf{v}}}_{i}| \,{\alpha }_{i}={\mathrm{soft}}\,{\mathrm{max}}\,({\mathrm{atten}}\,({{\bf{v}}}_{q},{{\bf{v}}}_{i}))\},\,{\boldsymbol{\alpha }}={E}_{c}^{{\rm{i}}{\rm{m}}{\rm{g}}}({{\bf{v}}}_{q},\{{{\bf{v}}}_{i}\})$$

These retrieved embeddings ${\widetilde{{\bf{V}}}}_{q}$ are passed into a fusion block, an 8-layer Transformer^50^ designed to iteratively integrate relevant information and refine representations:

2 $${\widehat{{\bf{V}}}}_{q}={\mathrm{Fusion}}_{{\mathrm{img}}}({\widetilde{{\bf{V}}}}_{q})$$

After fusion, we aggregate the outputs into a single enhanced image embedding ${\widehat{{\bf{v}}}}_{q}$ using a weighted sum based on the original attention weights:

3 $${\widehat{{\bf{v}}}}_{q}=\mathop{\sum }\limits_{i=1}^{n}{\alpha }_{i}\cdot {\widehat{{\bf{v}}}}_{i},\,{\widehat{{\bf{v}}}}_{i}\in {\widehat{{\bf{V}}}}_{q}$$

#### Knowledge arm: cross-modality retrieval and fusion

In parallel, we retrieve knowledge representations relevant to the query image. Each class *c* is associated with a set of domain-specific knowledge entries {**k**_1_,…,**k**_*m*_} encoded by a pre-trained text encoder. A class-specific expert ${E}_{c}^{know}$ compares the query image embedding **v**_*q*_ with each knowledge embedding and computes attention scores:

4 $${\widetilde{{\bf{K}}}}_{q}=\{{{\bf{k}}}_{i}| \,{\beta }_{i}={\mathrm{soft}}\,{\mathrm{max}}\,({\mathrm{atten}}({{\bf{v}}}_{q},{{\bf{k}}}_{i}))\},\,{\boldsymbol{\beta }}={E}_{c}^{{\rm{k}}{\rm{n}}{\rm{o}}{\rm{w}}}({{\bf{v}}}_{q},\{{{\bf{k}}}_{i}\})$$

These retrieved knowledge embeddings ${\widetilde{{\bf{K}}}}_{q}$ are also passed through another 8-layer Transformer fusion block to model relationships and refine semantic features:

5 $${\widehat{{\bf{K}}}}_{q}={{\mathrm{Fusion}}}_{{\mathrm{know}}}({\widetilde{{\bf{K}}}}_{q})$$

The final enhanced knowledge embedding ${\widehat{{\bf{k}}}}_{q}$ is obtained by attention-weighted aggregation:

6 $${\widehat{{\bf{k}}}}_{q}=\mathop{\sum }\limits_{i=1}^{m}{\beta }_{i}\cdot {\widehat{{\bf{k}}}}_{i},\,{\widehat{{\bf{k}}}}_{i}\in {\widehat{{\bf{K}}}}_{q}$$

#### Enhanced representation and learning objective

We align the enhanced image embedding ${\widehat{{\bf{v}}}}_{q}$ and enhanced knowledge embedding ${\widehat{{\bf{k}}}}_{q}$ using knowledge-enhanced contrastive learning. Our approach implements two types of supervision, which consists of local contrastive loss and global contrastive loss.

First, we use local contrastive loss to ensure precise matching between enhanced image and knowledge embeddings within each class. This helps the model learn class-specific patterns and characteristics. Second, we apply global contrastive loss to promote instance-level discriminability across the entire batch, encouraging the model to distinguish between different samples and different classes.

The total loss function is written as follows:

7 $${{\mathcal{L}}}_{{\mathrm{total}}}={{\mathcal{L}}}_{{\mathrm{local}}}+\lambda {{\mathcal{L}}}_{{\mathrm{global}}}$$

where *λ* is the balance parameter.

#### Local contrastive loss

The local contrastive loss aligns correct pairs of enhanced image and knowledge embeddings within each example. This helps the model learn class-specific patterns and characteristics. Suppose the total class number is *C*. For each query sample, we retrieve images and knowledge entries for each class, and obtain *C* enhanced image embeddings $\{{\widehat{{\bf{v}}}}_{1},\ldots ,{\widehat{{\bf{v}}}}_{C}\}$ and *C* enhanced knowledge embeddings $\{{\widehat{{\bf{k}}}}_{1},\ldots ,{\widehat{{\bf{k}}}}_{C}\}$.

Given a batch size *B* and a total of *C* classes, let the ground-truth class of sample *b* be *y*_*b*_ ∈ {1,…,*C*}. For that sample *b*, we retrieve images and knowledge entries for each class, and obtain *C* enhanced image embeddings $\{{\widehat{{\bf{v}}}}_{b,1},\ldots ,{\widehat{{\bf{v}}}}_{b,C}\}$ and *C* enhanced knowledge embeddings $\{{\widehat{{\bf{k}}}}_{b,1},\ldots ,{\widehat{{\bf{k}}}}_{b,C}\}$. ${\widehat{{\bf{v}}}}_{b,{y}_{b}}$ is the ground-truth enhanced image embedding and ${\widehat{{\bf{k}}}}_{b,{y}_{b}}$ is the ground-truth enhanced knowledge embedding.

We compute the pairwise similarity between all enhanced image and knowledge embeddings, and obtain a similarity matrix ${S}_{b}\in {{\mathbb{R}}}^{C\times C}$ with

$${S}_{b}(i,j)={\rm{s}}{\rm{i}}{\rm{m}}\,({\widehat{{\bf{v}}}}_{b,i},\,{\widehat{{\bf{k}}}}_{b,j}),\,i,j\in \{1,\ldots ,C\}.$$

Among all *C*^2^ pairs, only the diagonal entry corresponding to the ground-truth class (*i*, *j*) = (*y*_*b*_, *y*_*b*_) is considered positive; the remaining *C*^2^ − 1 entries act as negatives:

8 $${{\mathcal{L}}}_{local}^{(b)}=-\log \frac{\exp ({S}_{b}({y}_{b},{y}_{b})/\tau )}{{\sum }_{i=1}^{C}{\sum }_{j=1}^{C}\exp ({S}_{b}(i,j)/\tau )}.$$

The local loss is averaged over the batch:

$${{\mathcal{L}}}_{{\mathrm{local}}}=\frac{1}{B}\mathop{\sum }\limits_{b=1}^{B}{{\mathcal{L}}}_{{\mathrm{local}}}^{(b)}.$$

#### Global contrastive loss

While the local loss aligns individual samples, it lacks global structure awareness. Thus, we introduce the global contrastive loss. The global contrastive loss promotes instance-level discriminability across the entire batch. This encourages the model to distinguish between different samples and different classes. The loss compares each sample to all other samples in the batch.

We first introduce the image-to-knowledge direction loss. Let the set of all knowledge embeddings in the batch be ${\mathcal{K}}={\{{\widehat{{\bf{k}}}}_{q,c}\}}_{q=1,c=1}^{B,C}$ (*BC* items in total).

For sample *b*, we only use its ground-truth image embedding ${\widehat{{\bf{v}}}}_{b,{y}_{b}}$ to match only the ground-truth knowledge embedding ${\widehat{{\bf{k}}}}_{b,{y}_{b}}$ among all knowledge embeddings in the batch. All others are treated as negatives.

To avoid trivial matches, we mask out knowledge embeddings that come from another sample but share the same class as sample *b*:

$${{\mathcal{M}}}_{b}=\{(q,{y}_{b})\,| q\ne b\}.$$

Thus, the loss for the image-to-knowledge direction is:

9 $${{\mathcal{L}}}_{{\mathrm{img}}\to {\mathrm{know}}}^{(b)}=-\log \frac{\exp ({\mathrm{sim}}({\widehat{{\bf{v}}}}_{b,{y}_{b}},{\widehat{{\bf{k}}}}_{b,{y}_{b}})/\tau )}{{\sum }_{(q,c)\notin {{\mathcal{M}}}_{b}}\exp ({\mathrm{sim}}({\widehat{{\bf{v}}}}_{b,{y}_{b}},{\widehat{{\bf{k}}}}_{q,c})/\tau )}.$$

Similarly, for the knowledge-to-image direction:

10 $${{\mathcal{L}}}_{{\mathrm{know}}\to {\mathrm{img}}}^{(b)}=-\log \frac{\exp ({\mathrm{sim}}({\widehat{{\bf{k}}}}_{b,{y}_{b}},{\widehat{{\bf{v}}}}_{b,{y}_{b}})/\tau )}{{\sum }_{(q,c)\notin {{\mathcal{M}}}_{b}}\exp ({\mathrm{sim}}({\widehat{{\bf{k}}}}_{b,{y}_{b}},{\widehat{{\bf{v}}}}_{q,c})/\tau )}.$$

Both directions are averaged across the batch:

$${{\mathcal{L}}}_{{\mathrm{img}}\to {\mathrm{know}}}=\frac{1}{B}\mathop{\sum }\limits_{b=1}^{B}{{\mathcal{L}}}_{{\mathrm{img}}\to {\mathrm{know}}}^{(b)},\,{{\mathcal{L}}}_{{\mathrm{know}}\to {\mathrm{img}}}=\frac{1}{B}\mathop{\sum }\limits_{b=1}^{B}{{\mathcal{L}}}_{{\mathrm{know}}\to {\mathrm{img}}}^{(b)}.$$

The final global contrastive loss is computed by averaging across both directions:

11 $${{\mathcal{L}}}_{{\mathrm{global}}}=\frac{1}{2}({{\mathcal{L}}}_{{\mathrm{img}}\to {\mathrm{know}}}+{{\mathcal{L}}}_{{\mathrm{know}}\to {\mathrm{img}}})$$

#### Model inference

During inference, we process a query image through both arms to generate predictions. For each candidate class, we retrieve images and knowledge from our database. For each query image, we generate *C* enhanced image embeddings and *C* enhanced knowledge embeddings through the image and knowledge arms, where *C* is the number of candidate classes. For sample *b*, we compute *C* similarity scores by pairing the *C* enhanced image embeddings with the corresponding *C* enhanced knowledge embeddings. We calculate similarity for pairs $({\widehat{{\bf{v}}}}_{b,i},{\widehat{{\bf{k}}}}_{b,i})$ where *i* ∈ {1,…,*C*}. The similarity serves as the score for that class. The predicted label is assigned as the class with the highest similarity score.

#### Statistical analysis

Statistical significance between model performances was assessed using paired two-sided Student’s *t* tests. Statistical significance levels were denoted as follows: *p<0.05, **p<0.01, ***p<0.005. Confidence intervals (95% CI) were computed using bootstrap resampling. Random baseline performance was established using majority class prediction, where the model always predicts the most frequent class in each dataset.

### Patch-level image classification experiments

For patch-level evaluation, we excluded disease categories with fewer than 3 images, resulting in 40 melanocytic neoplasm classes for multi-class evaluation. We partitioned our curated dataset of pathology images into training (70%, 2,019 images), validation (15%, 431 images), and test (15%, 435 images) sets using a stratified split. For binary classification, we mapped the 44 WHO categories to melanoma versus nonmelanoma using MPATH-Dx assessment tools.

We compared our Melan-Dx framework against three baseline approaches (zero-shot, linear finetuned and fully finetuned foundation models) across four foundation model backbones (PLIP, PathGen, CONCH, and MUSK). For zero-shot baselines, we used pre-trained models with a text template “An H&E image of {*keyword*}” where keyword is the disease name and computed image-text similarity. For linear finetuned baselines, we added a linear layer on frozen vision encoders and trained only the linear layer. For fully finetuned baselines, we added a linear layer on frozen vision encoders and trained the entire models.

To assess clinical utility, we implemented a hierarchical accuracy metric that assigns partial credit based on the WHO classification hierarchy. This structure-aware evaluation recognizes that misclassifications within the same diagnostic family (e.g., different types of nevi) are less clinically significant than misclassifications across major categories (e.g., confusing melanoma with benign lesions).

We used AdamW^51^ optimizer with grid search over learning rates {1 × 10^−3^, 1 × 10^−4^, 1 × 10^−5^} and weight decay of 1 × 10^−2^ for linear finetuned, fully finetuned, and Melan-Dx methods. For Melan-Dx, we set the balance parameter between local and global losses *λ* = 0.01 for 40-class classification. For binary classification, we set *λ* = 0.75 for CONCH+Melan-Dx, *λ* = 0.9 for MUSK+Melan-Dx, and *λ* = 1.0 for others. Training used maximum 100 epochs with early stopping at 20 epochs of no validation improvement. For training time comparison experiments, we used 30 epochs with learning rate 1 × 10^−4^ and recorded training time. All experiments were conducted on a single A100 80GB GPU.

### Melan-Dx outperforms parameter-efficient fine-tuning

To evaluate whether explicit knowledge integration outperforms implicit parameter adaptation, we compared Melan-Dx against Low-Rank Adaptation (LoRA)^52^ fine-tuning on the 40-class classification task. LoRA is a parameter-efficient method that introduces trainable rank decomposition matrices into specific layers while keeping pretrained weights frozen. We applied LoRA with rank *r* = 8, alpha *α* = 16, and dropout rate 0.1 to the attention projection layers: for PLIP, CONCH, and MUSK, we fine-tuned the query, key, and value projections. Due to PathGen uses merged QKV projections in its OpenCLIP implementation, we fine-tuned the output projection layer instead. All other training configurations remained identical to our baseline experiments.

Melan-Dx consistently outperformed LoRA fine-tuning across all evaluation metrics and foundation models (Supplementary Fig. S3). For Top-1 accuracy, Melan-Dx achieved substantial improvements: PLIP+Melan-Dx reached 0.598 versus 0.457 for LoRA (*P* = 5.57 × 10^−47^), PathGen + Melan-Dx 0.648 versus 0.547 (*P* = 2.35 × 10^−33^), CONCH+Melan-Dx 0.602 versus 0.494 (*P* = 2.77 × 10^−32^), and MUSK+Melan-Dx 0.699 versus 0.566 (*P* = 1.86 × 10^−45^), representing 10.1–14.1% absolute improvements. For Top-3 accuracy, similar patterns emerged with Melan-Dx showing gains across PLIP (0.784 vs. 0.708, *P* = 4.77 × 10^−31^), CONCH (0.832 vs. 0.789, *P* = 5.69 × 10^−20^), and MUSK (0.874 vs. 0.841, *P* = 9.28 × 10^−14^), while PathGen achieved comparable performance (0.816 vs. 0.818, *P* = 5.00 × 10^−1^). The hierarchical accuracy metric further demonstrated Melan-Dx’s advantages: PLIP (0.636 vs. 0.525, *P* = 6.15 × 10^−38^), PathGen (0.690 vs. 0.599, *P* = 2.87 × 10^−36^), CONCH (0.647 vs. 0.553, *P* = 9.02 × 10^−38^), and MUSK (0.729 vs. 0.612, *P* = 4.33 × 10^−47^), showing 9.1–11.7% improvements across all backbones. For weighted F1 score, Melan-Dx also showed consistent improvements: PLIP (0.592 vs. 0.426, *P* = 7.83 × 10^−55^), PathGen (0.639 vs. 0.530, *P* = 3.36 × 10^−38^), CONCH (0.592 vs. 0.479, *P* = 2.15 × 10^−37^), and MUSK (0.689 vs. 0.570, *P* = 2.02 × 10^−37^).

These results demonstrate that explicit knowledge integration through retrieval and fusion mechanisms works better than parameter-efficient adaptation for fine-grained melanocytic neoplasm classification. While LoRA adapts the vision encoder’s internal representations through trainable matrices, Melan-Dx preserves the rich pretrained features while augmenting them with structured medical knowledge retrieved from our curated atlas.

### Analysis of classification cases

To understand the limitations of our approach, we analyzed misclassification cases. Supplementary Fig. S4 shows a representative failure case where Melan-Dx incorrectly predicted “Low-CSD melanoma (including superficial spreading melanoma)” for a dysplastic nevus query image. Our system retrieves two reference images for each disease class and makes predictions based on all retrieved information. Here we visualize the retrieval results for the ground truth class (dysplastic nevus), which obtained retrieval scores of 0.5776 and 0.4224. This suggests that for complex cases, the retrieved examples from the correct category may not provide sufficient visual support, while retrieved images from other classes might show misleading similarities. Such misclassifications may occur when the query image exhibits atypical or complex histological patterns that are not well-represented in our atlas.

In contrast, successful cases like Fig. 4e demonstrate the importance of retrieval quality. For the correctly classified example of junctional, compound, and dermal naevi (Fig. 4e), the retrieved images show strong morphological correspondence with retrieval scores of 0.5602 and 0.4398, which indicates that the pretrained encoder effectively captured the relevant histological patterns and retrieved visually similar examples from our atlas. This comparison highlights that retrieval quality is highly dependent on whether the query image’s features align well with examples in our atlas. These findings indicate that improving retrieval performance and expanding the dataset with more diverse examples could help address challenging cases.

### Whole slide image classification experiments on HISTAI skin dataset

We extended Melan-Dx to WSI classification using the HISTAI skin dataset for binary melanoma versus nonmelanoma classification. The HISTAI skin dataset does not contain predefined labels, but each case includes a diagnostic result field in its metadata. We filtered the dataset using GPT-4o to match only cases with clear melanoma and nonmelanoma diagnoses. After two rounds of strict matching, we retained consistently matched cases. Only cases with explicit diagnoses were retained, followed by manual verification by the authors to confirm correctness. We randomly selected 1000 WSIs from this filtered dataset for experiments, including 500 melanoma and 500 nonmelanoma cases.

Our WSI processing pipeline divides each slide into 512 × 512 pixel patches at ×10 magnification. Individual patches are processed through either baseline foundation models or our Melan-Dx-enhanced framework to generate patch-level embeddings. These patch-level representations are aggregated using ABMIL^44^ to produce slide-level predictions.

We partitioned the dataset into training (70%, 700 WSIs), validation (15%, 150 WSIs), and test (15%, 150 WSIs) sets using a stratified split to maintain balanced representation. For all WSI experiments, including both few-shot and supervised learning, we kept the same validation and test sets across different training scenarios. To ensure fair comparison, we removed 27 duplicate WSIs from the training set.

We used the AdamW^51^ optimizer with a weight decay of 1 × 10^−2^. Hyperparameter optimization was conducted across learning rates {1 × 10^−2^, 1 × 10^−3^, 1 × 10^−4^, 1 × 10^−5^, 1 × 10^−6^} for maximum 100 epochs with early stopping at 20 epochs of no validation improvement.

We conducted comprehensive few-shot learning experiments to assess data efficiency using varying numbers of training samples per class (2, 8, 32, 64, 96, and 128 shots). This is critical given the limited availability of annotated WSI data in specialized medical domains. We also performed supervised learning experiments using the complete training set. We compared Melan-Dx-enhanced models against baseline foundation models on binary WSI classification tasks. In addition to ABMIL aggregation, we further evaluated Melan-Dx with TITAN aggregation and TITAN with CONCH v1.5 under the same experimental settings (Supplementary Fig. S5). All experiments were conducted on a single Nvidia A100 PCIe GPU (80GB memory) or Nvidia H200 NVL GPU (141 GB memory).

### Whole slide image classification experiments on the SOPHIE dataset

To further validate the effectiveness of our approach, we evaluated Melan-Dx on the SOPHIE^53^ dataset, an independent external melanoma diagnostic dataset containing 51 WSIs with binary labels (melanoma vs. nevi). We randomly selected 40% of WSIs (20 slides) as the test set and used the remaining 60% (31 slides) for training, repeating this data split five times to ensure robust evaluation. Each WSI was divided into 224 × 224 pixel patches at 10 × magnification. We used Adam^54^ optimizer with learning rate grid search over {1 × 10^−2^, 1 × 10^−3^, 1 × 10^−4^, 1 × 10^−5^, 1 × 10^−6^} for maximum 100 epochs. Since MUSK model showed the best patch-level performance in previous experiments, we use MUSK backbone for this experiment.

Melan-Dx demonstrated consistent improvements over the MUSK baseline on the SOPHIE dataset across all evaluation metrics (Supplementary Fig. S6). MUSK+Melan-Dx achieved 0.951 ROC AUC compared to 0.917 for MUSK baseline, 0.890 accuracy versus 0.870 for MUSK, 0.940 AUPRC versus 0.904 for MUSK, 0.889 F1 score versus 0.869 for MUSK, 0.900 precision versus 0.883 for MUSK, and 0.882 specificity versus 0.873 for MUSK.

## Supplementary information

Supplementary Information

## Data Availability

Images and knowledge from Penn Melan-Dx knowledge atlas is a proprietary database at the University of Pennsylvania. Source code, along with image and knowledge embeddings, are available at https://www.github.com/zhihuanglab/Melan-Dx-code. Raw region-of-interest images and knowledge descriptions are available upon written request with an approved material transfer agreement.
