# Supplementary material for: Melan-Dx: a knowledge-enhanced vision-language framework improves differential diagnosis of melanocytic neoplasm pathology
Source: NPJ Digit Med. 2026 Jan 20;9:171. doi: 10.1038/s41746-026-02357-3 (PMC12914046; doi:10.1038/s41746-026-02357-3)
Supplement: Supplementary file 1 — Supplementary Information [file 41746_2026_2357_MOESM1_ESM.pdf]

Supplementary Information for

Melan-Dx: A knowledge-enhanced vision-language framework improves differential diagnosis of melanocytic neoplasm pathology

Supplementary Figures

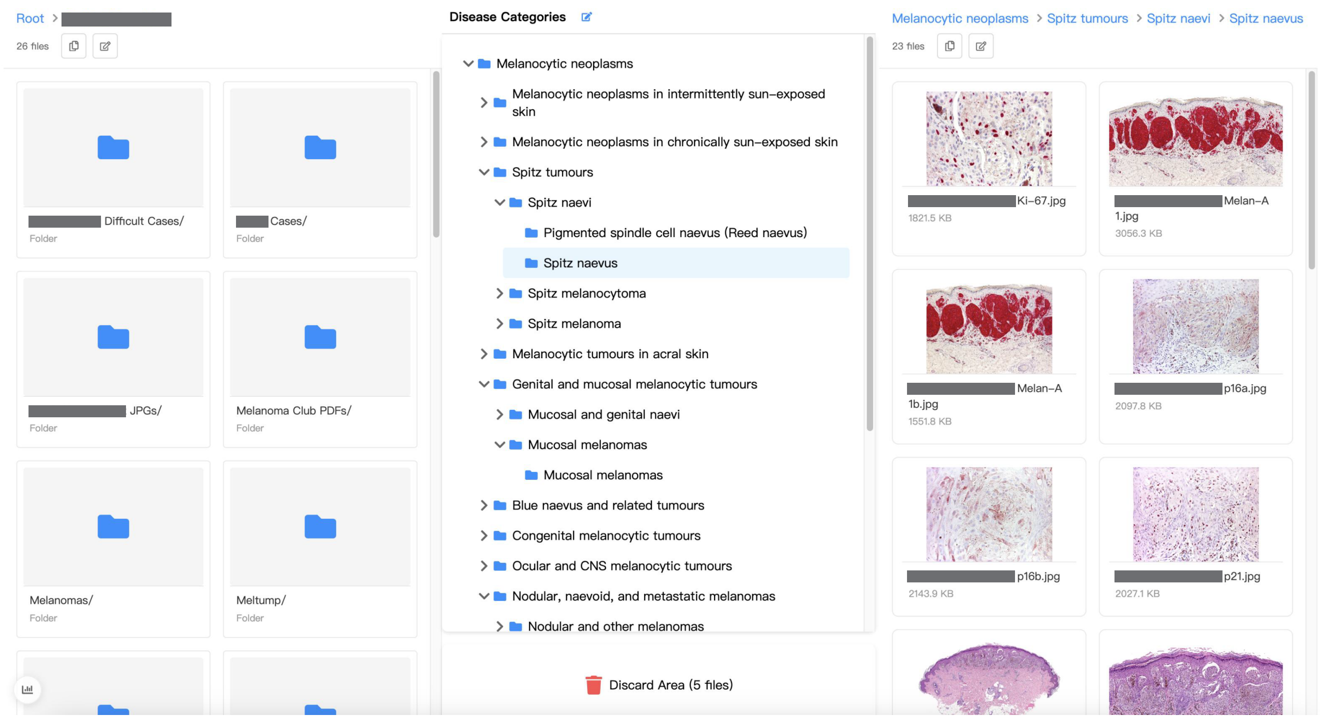

Supplementary Figure S1. Web-based annotation interface for expert dermatopathologist review. The interface consists of three panels: the left panel shows raw image folders, the center panel displays the hierarchical WHO classification taxonomy for melanocytic tumors, and the right panel presents visual thumbnails of images assigned to each disease category.

Level 1	Level 2	Level 3
Nodular, naevoid, and metastatic melanomas (132; 4.56%)	Nodular and other melanomas (103; 78.03%)	Dermal melanoma (11; 10.68%)
		Naevoid melanoma (51; 49.51%)
		Nodular melanoma (41; 39.81%)
	Metastatic melanomas (29; 21.97%)	Melanoma metastatic to other organs (20; 68.97%)
		Melanoma metastatic to the skin (9; 31.03%)
Blue naevus and related tumours (89; 3.08%)	Blue naevi and melanocytoses (66; 74.16%)	Blue naevus (58; 87.88%)
		Congenital dermal melanocytosis (6; 9.09%)
		Naevus of Ito and naevus of Ota (2; 3.03%)
	Melanomas arising from blue naevi (23; 25.84%)	Melanoma arising in blue naevus (23; 100.00%)
Melanocytic neoplasms in intermittently sun-exposed skin (1590; 54.96%)	Naevi (703; 44.21%)	Combined naevus (122; 17.35%)
		Recurrent naevus (58; 8.25%)
		Meyerson naevus (2; 0.28%)
		Halo naevus (55; 7.82%)
		Special-site naevi (of the breast, axilla, scalp, and ear) (14; 1.99%)
		Naevus spilus (3; 0.43%)
		Dysplastic naevus (242; 34.42%)
		Simple lentigo and lentiginous melanocytic naevus (35; 4.98%)
		Junctional, compound, and dermal naevi (172; 24.47%)
	Melanocytomas (188; 11.82%)	MITF pathway-activated melanocytic tumours (8; 4.26%)
		BAP1-inactivated melanocytoma (4; 2.13%)
		Pigmented epithelioid melanocytoma (87; 46.28%)
		WNT-activated deep penetrating/plexiform melanocytoma (naevus) (89; 47.34%)
	Melanoma intermittently sun-exposed skin (699; 43.96%)	Low-CSD melanoma (including superficial spreading melanoma) (699; 100.00%)
Melanocytic neoplasms in chronically sun-exposed skin (434; 15.00%)	Melanoma in chronically sun-exposed skin (434; 100.00%)	Desmoplastic melanoma (179; 41.24%)
		Lentigo maligna melanoma (255; 58.76%)
Spitz tumours (311; 10.75%)	Spitz naevi (184; 59.16%)	Spitz naevus (114; 61.96%)
		Pigmented spindle cell naevus (Reed naevus) (70; 38.04%)
	Spitz melanocytoma (57; 18.33%)	Spitz melanocytoma (Atypical Spitz tumour) (57; 100.00%)
	Spitz melanoma (70; 22.51%)	Spitz melanoma (70; 100.00%)
Genital and mucosal melanocytic tumours (50; 1.73%)	Mucosal and genital naevi (8; 16.00%)	Genital naevus (6; 75.00%)
		Melanosis (2; 25.00%)
	Mucosal melanomas (42; 84.00%)	Mucosal melanomas (42; 100.00%)
Congenital melanocytic tumours (115; 3.98%)	Congenital naevi (86; 74.78%)	Proliferative nodules in congenital melanocytic naevus (46; 53.49%)
		Congenital melanocytic naevus (40; 46.51%)
	Melanomas arising in congenital naevi (29; 25.22%)	Melanoma arising in giant congenital naevus (29; 100.00%)
Melanocytic tumours in acral skin (124; 4.29%)	Acral naevi (32; 25.81%)	Acral naevus (32; 100.00%)
	Acral melanomas (92; 74.19%)	Acral melanoma (92; 100.00%)
Ocular and CNS melanocytic tumours (48; 1.66%)	Conjunctival melanocytic tumours (24; 50.00%)	Conjunctival melanoma (11; 45.83%)
		Conjunctival melanocytic intraepithelial lesions (7; 29.17%)
		Conjunctival naevus (6; 25.00%)
	Uveal melanocytic tumours (8; 16.67%)	Uveal melanoma (2; 25.00%)
		Uveal melanocytoma (6; 75.00%)
	CNS melanocytic tumours (16; 33.33%)	Circumscribed meningeal melanocytic neoplasms: melanocytoma and melanoma (11; 68.75%)
		Diffuse meningeal melanocytic neoplasms: melanocytosis and melanomatosis (5; 31.25%)

Supplementary Figure S2. Hierarchical organization of the Melan-Dx dermatopathology atlas following the WHO classification system. The three-tier taxonomy comprises Level 1 (9 root categories), Level 2 (20 intermediate clinical classifications), and Level 3 (44 specific diagnostic entities). Numbers in parentheses indicate image count and percentage of parent category.

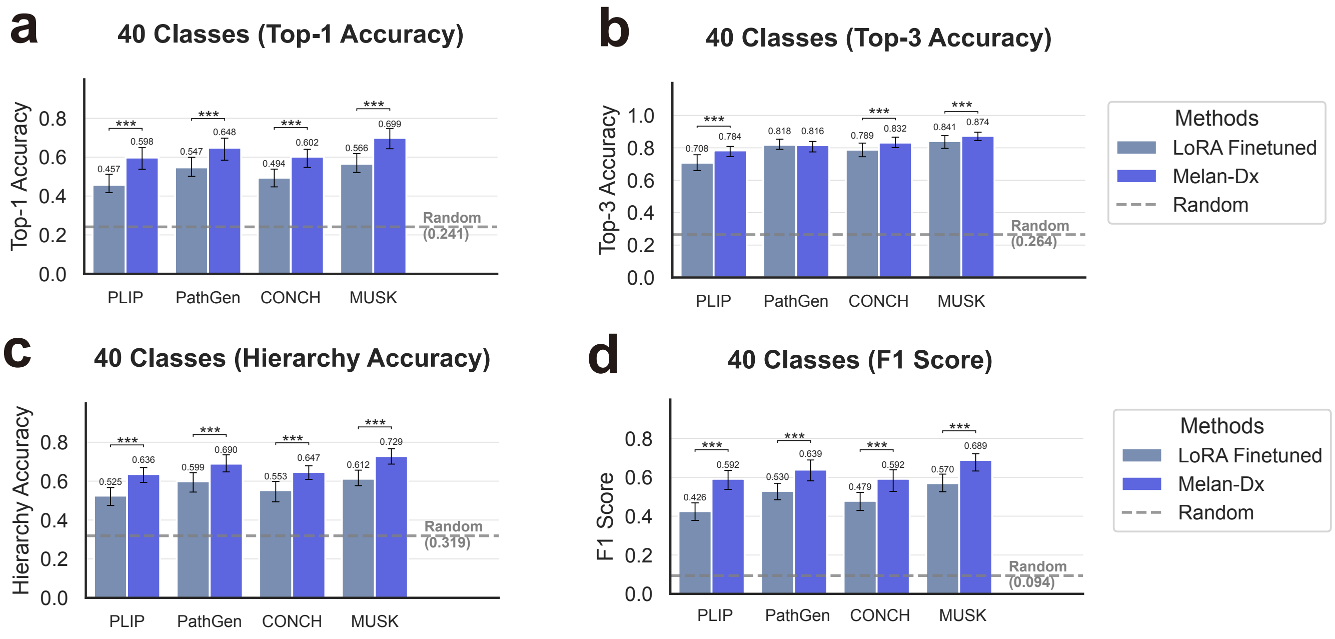

Supplementary Figure S3. Comparison with LoRA fine-tuning on 40-class classification. Melan-Dx versus LoRA fine-tuning across four foundation models for (a) Top-1 accuracy, (b) Top-3 accuracy, and (c) hierarchical accuracy, and (d) F1 score. Error bars represent 95% confidence intervals. Statistical significance was assessed using paired two-sided Student's t-test: *p < 0.05, **p < 0.01, ***p < 0.005.

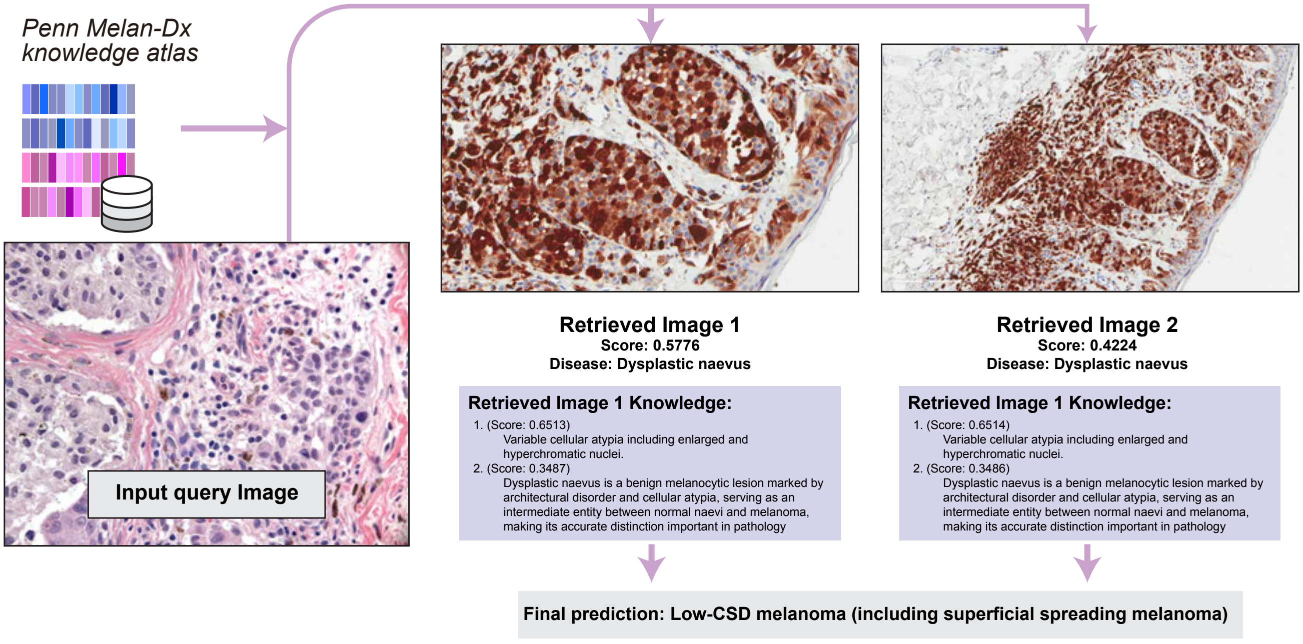

Supplementary Figure S4. Example of misclassification case. The ground truth diagnosis is "dysplastic naevus", while the model incorrectly predicted "Low-CSD melanoma (including superficial spreading melanoma)". The retrieved support images for the ground truth class received scores of 0.5776 and 0.4224, indicating limited visual support in complex histological contexts.

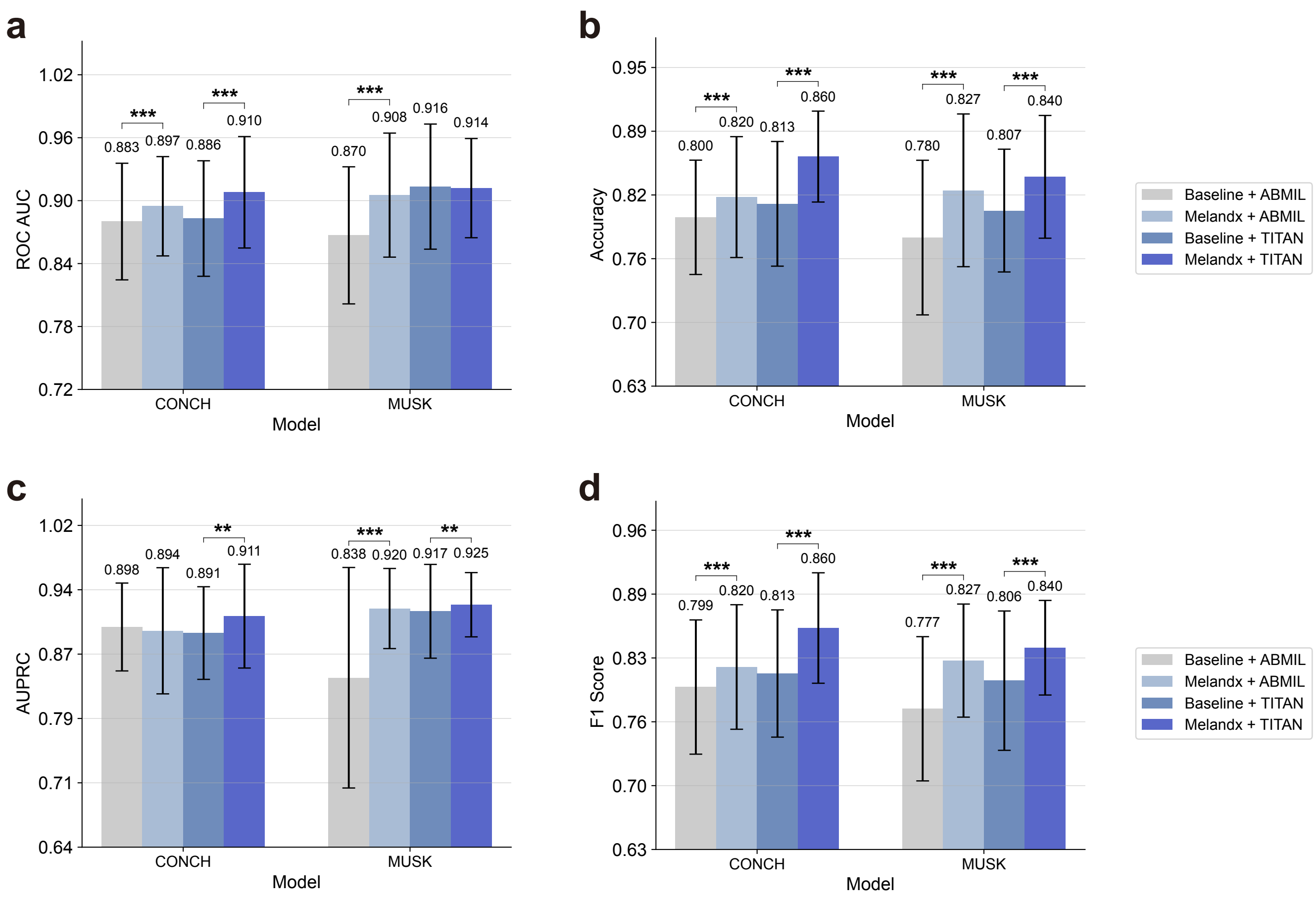

e

Method	ROC AUC	Accuracy	AUPRC	F1 Score
TITAN (CONCH v1.5)	0.917	0.834	0.924	0.834

Supplementary Figure S5. Performance comparison of Melan-Dx on the HISTAI skin dataset. Evaluation of CONCH v1.0 and MUSK backbones with ABMIL and TITAN aggregation, with and without Melan-Dx enhancement, on (a) ROC AUC, (b) accuracy, (c) AUPRC, and (d) F1 score. (e) Performance of TITAN with CONCH v1.5 on the HISTAI dataset. Error bars represent 95% confidence intervals. Statistical significance: *$p < 0.05$, **$p < 0.01$, ***$p < 0.005$ (paired two-sided Student's t-test).

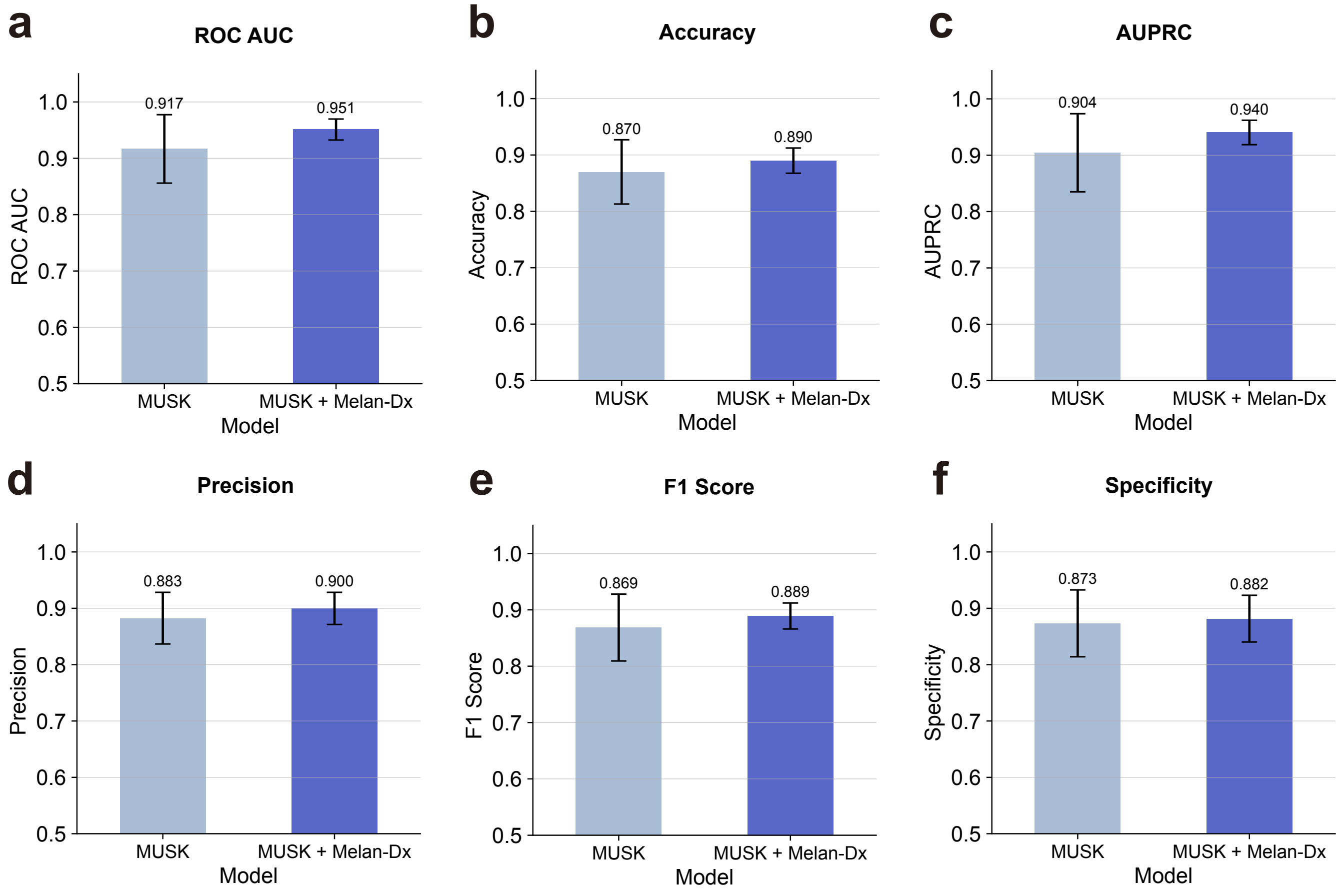

Supplementary Figure S6. WSI-level performance on the SOPHIE dataset. Performance comparison of TITAN, MUSK baseline, and MUSK+Melan-Dx for (a) ROC AUC, (b) accuracy, (c) AUPRC, (d) precision, (e) F1 score, and (f) specificity on binary melanoma vs. nevi classification. Error bars represent standard deviation across data splits.

Supplementary Tables

Supplementary Table S1. Notation summary used in this paper.

Notation	Description
I_q	Input query image
$\mathbf{v}_q$	Image embedding from vision encoder
$\mathscr{A} = \{\mathscr{I}_c, \mathscr{K}_c\}_{c=1}^{C}$	Penn Melan-Dx Knowledge Atlas
$\mathscr{I}_c$	Image set for class c in the atlas
$\mathscr{K}_c$	Knowledge set for class c in the atlas
$\mathbf{v}_i$	i-th image embedding in $\mathscr{I}_c$
$\mathbf{k}_i$	i-th knowledge embedding in $\mathscr{K}_c$
E_c^{img}	Image expert module for class c
E_c^{know}	Knowledge expert module for class c
$\tilde{\mathbf{V}}_q$	Retrieved visual features
$\tilde{\mathbf{K}}_q$	Retrieved knowledge features
$\hat{\mathbf{V}}_q$	Fused visual features
$\hat{\mathbf{K}}_q$	Fused knowledge features
$\hat{\mathbf{v}}_q$	Enhanced image embedding
$\hat{\mathbf{k}}_q$	Enhanced knowledge embedding
s_c	Similarity score for class c
$\mathscr{L}_{\text{local}}$	Local contrastive loss
$\mathscr{L}_{\text{global}}$	Global contrastive loss
λ	Balance weight between two losses
B	Batch size
C	Number of disease classes
**Top 1**, **Top 2**	Top-1 and Top-2 retrieved visual samples
K_{A1}, K_{A2}	Retrieved knowledge entries from Class A

Supplementary Algorithms

Algorithm 1 Melan-Dx Training

Input: Training set $\{(I_q^{(b)}, y^{(b)})\}_{b=1}^{B}$; frozen encoders f_v, f_t; Penn Melan-Dx Knowledge Atlas $\mathscr{A} = \{\mathscr{I}_c, \mathscr{K}_c\}_{c=1}^{C}$; top-$k$, top-$m$; balance λ

Encode all atlas images and knowledge texts using f_v and f_t, and store embeddings by class

while not converged **do**

  Sample a minibatch of query images $\{I_q^{(b)}\}$

  for each query image I_q **do**

    Extract visual feature $\mathbf{v}_q = f_v(I_q)$

    for each class $c = 1 \ldots C$ **do**

      Image arm:

        Compute attention $\alpha = E_c^{\text{img}}(\mathbf{v}_q, \mathscr{I}_c)$

        Retrieve top-k visual features $\tilde{\mathbf{V}}_q$ and fuse: $\hat{\mathbf{V}}_q = \text{Fusion}_{\text{img}}(\tilde{\mathbf{V}}_q)$

        Aggregate to get enhanced image embedding $\hat{\mathbf{v}}_q = \sum_i \alpha_i \hat{\mathbf{v}}_i$

      Knowledge arm:

        Compute attention $\beta = E_c^{\text{know}}(\mathbf{v}_q, \mathscr{K}_c)$

        Retrieve top-m knowledge features $\tilde{\mathbf{K}}_q$ and fuse: $\hat{\mathbf{K}}_q = \text{Fusion}_{\text{know}}(\tilde{\mathbf{K}}_q)$

        Aggregate to get enhanced knowledge embedding $\hat{\mathbf{k}}_q = \sum_i \beta_i \hat{\mathbf{k}}_i$

    end for

  end for

  Compute local loss $\mathscr{L}_{\text{local}}$ between $\hat{\mathbf{v}}_q$ and $\hat{\mathbf{k}}_q$ within each class

  Compute global loss $\mathscr{L}_{\text{global}}$ across the minibatch

  Total loss $\mathscr{L}_{\text{total}} = \mathscr{L}_{\text{local}} + \lambda \mathscr{L}_{\text{global}}$

  Update E_c^{img}, E_c^{know}, and fusion modules; keep f_v, f_t frozen

end while

Algorithm 2 Melan-Dx Inference

Input: Query image I_q; trained E_c^{img}, E_c^{know}; fusion modules; Penn Melan-Dx Knowledge Atlas $\mathscr{A} = \{\mathscr{I}_c, \mathscr{K}_c\}_{c=1}^{C}$

Extract feature $\mathbf{v}_q = f_v(I_q)$

for each class $c = 1 \ldots C$ **do**

  Compute $\alpha = E_c^{\text{img}}(\mathbf{v}_q, \mathscr{I}_c)$, retrieve $\tilde{\mathbf{V}}_q$, fuse $\hat{\mathbf{V}}_q = \text{Fusion}_{\text{img}}(\tilde{\mathbf{V}}_q)$, aggregate $\hat{\mathbf{v}}_q$

  Compute $\beta = E_c^{\text{know}}(\mathbf{v}_q, \mathscr{K}_c)$, retrieve $\tilde{\mathbf{K}}_q$, fuse $\hat{\mathbf{K}}_q = \text{Fusion}_{\text{know}}(\tilde{\mathbf{K}}_q)$, aggregate $\hat{\mathbf{k}}_q$

  Compute similarity $s_c = \text{sim}(\hat{\mathbf{v}}_q, \hat{\mathbf{k}}_q)$

end for

Predict $\hat{y} = \arg\max_c s_c$
